# CCNYL1, but Not CCNY, Cooperates with CDK16 to Regulate Spermatogenesis in Mouse

**DOI:** 10.1371/journal.pgen.1005485

**Published:** 2015-08-25

**Authors:** Zhenzhen Zi, Zhuzhen Zhang, Qingrun Li, Weiwei An, Liyong Zeng, Dayuan Gao, Ying Yang, Xueliang Zhu, Rong Zeng, Winnie Waichi Shum, Jiarui Wu

**Affiliations:** 1 Hefei National Laboratory for Physical Sciences at Microscale and School of Life Sciences, University of Science & Technology of China, Hefei, Anhui, China; 2 Key Laboratory of Systems Biology, Institute of Biochemistry and Cell Biology, Shanghai Institutes for Biological Sciences, Chinese Academy of Sciences, Shanghai, China; 3 State Key Laboratory of Cell Biology, Institute of Biochemistry and Cell Biology, Shanghai Institutes for Biological Sciences, Chinese Academy of Sciences, Shanghai, China; 4 School of Life Science and Technology, ShanghaiTech University, Shanghai, China; 5 Shanghai Advanced Research Institute, Chinese Academy of Sciences, Shanghai, China; University of Nevada School of Medicine, UNITED STATES

## Abstract

*Cyclin Y-like 1* (*Ccnyl1*) is a newly-identified member of the cyclin family and is highly similar in protein sequences to *Cyclin Y* (*Ccny*). However, the function of *Ccnyl1* is poorly characterized in any organism. Here we found that *Ccnyl1* was most abundantly expressed in the testis of mice and was about seven times higher than the level of *Ccny*. Male *Ccnyl1*-/- mice were infertile, whereas both male and female *Ccny*-/- mice displayed normal fertility. These results suggest that *Ccnyl1*, but not *Ccny*, is indispensable for male fertility. Spermatozoa obtained from *Ccnyl1*-/- mice displayed significantly impaired motility, and represented a thinned annulus region and/or a bent head. We found that the protein, but not the mRNA, level of cyclin-dependent kinase 16 (CDK16) was decreased in the testis of *Ccnyl1*-/- mice. Further study demonstrated that CCNYL1 interacted with CDK16 and this interaction mutually increased the stability of these two proteins. Moreover, the interaction increased the kinase activity of CDK16. In addition, we observed an alteration of phosphorylation levels of CDK16 in the presence of CCNYL1. We identified the phosphorylation sites of CDK16 by mass spectrometry and revealed that several phosphorylation modifications on the N-terminal region of CDK16 were indispensable for the CCNYL1 binding and the modulation of CDK16 kinase activity. Our results therefore reveal a previously unrecognized role of CCNYL1 in regulating spermatogenesis through the interaction and modulation of CDK16.

## Author Summary

Infertility is a global public health issue that affects up to 15% of reproductive-age couples worldwide, and male infertility contributes to about 50% of these cases. However, our knowledge of the genetic causes of infertility is still limited. Nowadays, the knockout or mutant animal models have become valuable tools for identifying dysfunctional genes in the infertile population. Here, we show that CCNYL1 is specifically and highly expressed in the testis and mainly localized on the plasma membrane of spermatocytes and spermatids. Using the *Ccnyl1* knockout model, we found that male but not female *Ccnyl1*-/- mice were infertile, accompanied by sperm defects in both motility and structural integrity. Most cyclins are known to function by forming complexes with CDKs, and our study shows for the first time that the partner for CCNYL1 is CDK16. We found that the interaction between CCNYL1 and CDK16 was indispensable for the stability and activity of CDK16. Phosphorylation modifications on CDK16 were also involved in this process. Our study thus reveals an important role of CCNYL1 in regulating male mouse fertility by cooperating with CDK16 and provides insights into the mechanisms underlying cases of male infertility with similar phenotypes.

## Introduction

Cyclins comprise a family of highly conserved proteins and exert their crucial roles by activating cyclin-dependent kinases (CDKs). The founding members, Cyclin A and Cyclin B, were discovered to be oscillated in their protein levels during the cell cycle, which is required to activate CDK1, and consequently influence the cell cycle [1]. However, some Cyclins and CDKs do not necessarily regulate the cell cycle but instead regulate transcription and other cellular processes. For instance, Cyclin H/CDK7, Cyclin C/CDK8, Cyclins T/K/CDK9, and Cyclin L/CDK11 partners are reported to be involved in modulating transcription and/or RNA splicing [2]. Additionally, CCNY, which is highly conserved among metazoa [3], associates with Eip63E (CDK14) in regulating larval development and metamorphosis in Drosophila without influencing cell cycle [4]. Recent studies have shown that CCNY also interacts with CDK16 in HEK293A cell lines, and male *Cdk16* knockout mice are infertile, implying a role for *Ccny* in male fertility [5]. In addition, in cultured cells and Xenopus embryos, CCNY/CDK14 mediates the binding and phosphorylation of LRP6, and regulates Wnt/β-catenin signaling in orchestrating G2/M cell cycle progression [6]. As well as these Cyclins/CDKs partners, there are also several "orphan" cyclins and "orphan" CDKs for which no interacting partners have been yet identified [3]. CCNYL1, a newly identified protein, is one of these "orphan" cyclins. It shows 79% similarity in protein sequences with CCNY, but its function remains elusive.

CDKs are serine/threonine kinases, first discovered in yeast, and found to promote transitions between different cell cycle stages [7]. The kinase activities of CDKs are modulated by interacting with the regulatory subunits of cyclins and CDK inhibitors. CDKs can also be phosphorylated by other protein kinases and dephosphorylated by phosphatases. The phosphorylation of these sites is also involved in regulating the kinase activity of CDKs [8–9]. Thus far, about 26 members of the CDK family have been identified. They possess high homology in their amino acid sequence, and many are named according to their highly conserved PSTAIRE motif, which is critical for the binding of Cyclin partners [10]. Like these Cyclins, CDKs are not only involved in cell cycle regulation, but also play essential roles in other biological processes, such as transcription and neuronal functions [11]. CDK16 was identified in 1992 [12], is a member of the PCTAIRE kinases, and has been shown to be particularly highly expressed in the testis and brain [13]. The kinase activity of CDK16 seems to be cell cycle-dependent in cell lines, but it remains uncertain if CDK16 plays a role in cell cycle regulation [14]. CDK16 is the substrate of protein kinase A, phosphorylation of Ser119 generates a binding site for 14-3-3, while phosphorylation of Ser153 controls the kinase activity [5, 15]. A number of studies show that CDK16 is involved in various biological processes, including neurite outgrowth [15], myoblast migration [16], exocytosis [17], vesicle transport [17–18] and spermatogenesis [5].

To address the uncharacterized functional role of CCNYL1 protein, in this study we used *Ccnyl1* gene disrupted mice. *Ccnyl1*-/- mice were overtly healthy, but males were infertile. In contrast, the knockout of *Ccny*, a homologue of *Ccnyl1*, displayed normal spermatogenesis and fertility, which indicates that *Ccnyl1*, but not *Ccny*, is essential for male fertility. Our study showed that CCNYL1 is highly expressed in the plasma membrane of spermatocytes and spermatids, and its deletion leads to impaired motility and defects in sperm structural integrity. Further investigations revealed that CCNYL1 is required for the interaction with CDK16, which results in mutually increased protein stability and strikingly increased CDK16 kinase activity. We also demonstrated that modifications of CDK16 phosphorylation play critical roles in interacting with CCNYL1 and regulating the kinase activity of CDK16. Overall, a novel functional role for CCNYL1 in spermatogenesis and male fertility has been identified.

## Results

### Temporal and Spatial Expression Profiles of CCNYL1 in Mice

**Fig 1:**
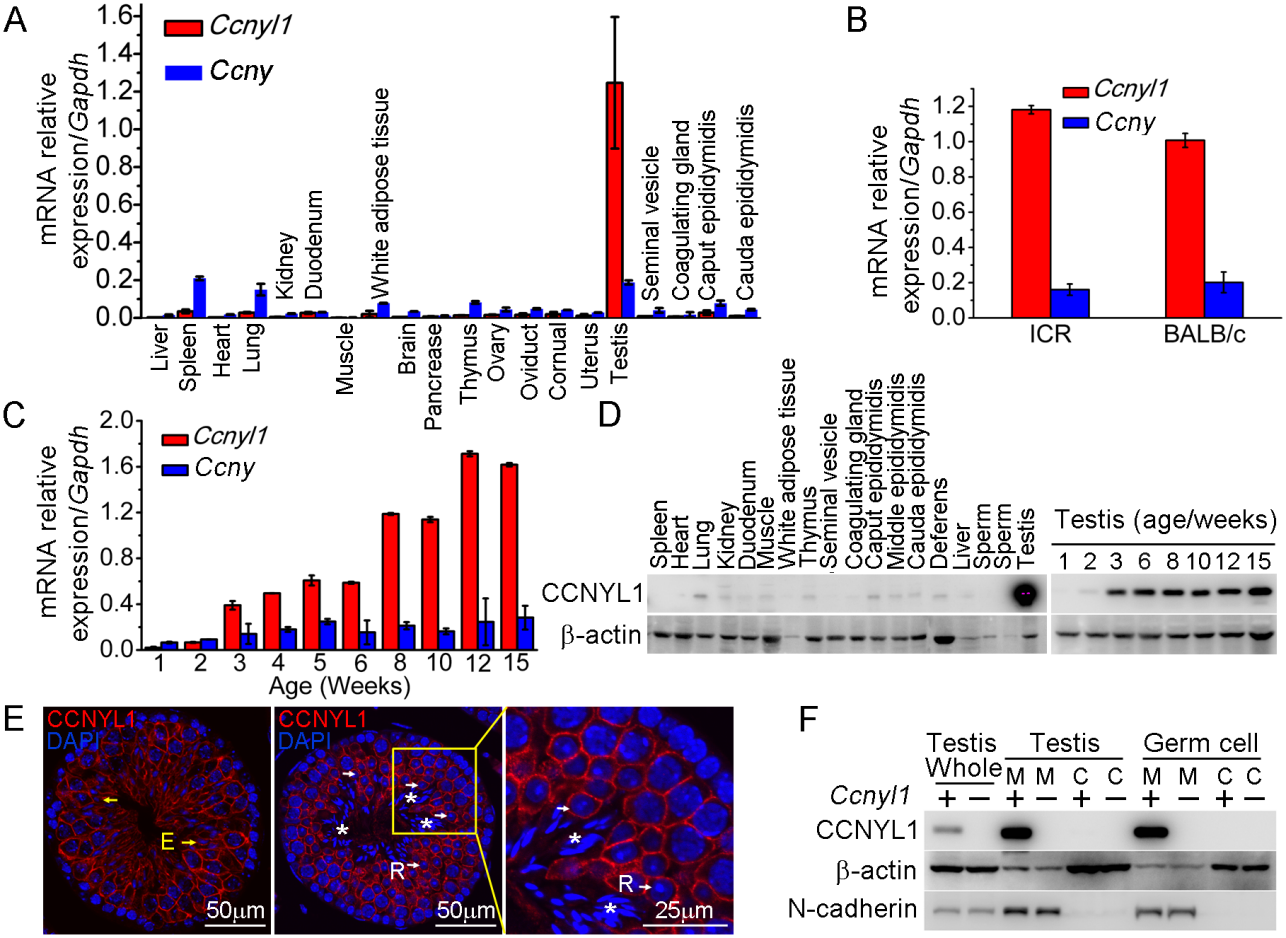
The expression pattern of CCNYL1 in mice. (A) *Ccnyl1* and *Ccny* mRNA levels were measured in different tissues of adult WT mice (n = 4). (B) *Ccnyl1* and *Ccny* mRNA levels were measured in testes of ICR and BABL/c mouse lines (n = 4 per group). (C) Both *Ccnyl1* and *Ccny* mRNA levels were measured in testes of WT mice of different age (n = 4). (D) CCNYL1 protein levels were measured in different tissues of adult WT mice, and in testes of WT mice of different ages. β-actin served as the protein loading control. (E) Immunolabeling of CCNYL1 (red) during the spermatogenic cycle of testicular sections. Nuclei were labeled with DAPI (blue). R: round spermatids (white arrows); E: elongated spermatids (yellow arrows); spermatozoa with shed residual bodies (asterisks). Scale bar for left and middle panels: 50 μm, scale bar for right panel: 25 μm. (F) CCNYL1 protein expression was measured in membrane extracts (designated as M) and cytoplasmic extracts (designated as C) isolated from testicular or germ cells of WT and *Ccnyl1-/-* mice. N-cadherin served as the membrane protein-loading control, whereas β-actin served as the cytoplasmic protein loading control. Data in bar graphs are presented as mean ± SEM.

Real-time PCR results showed that *Ccnyl1* was expressed at strikingly high levels in the testis, and was about seven times higher than that of its homologue, *Ccny* (Fig 1A), and similar results were also obtained from mice of other backgrounds, such as ICR (Fig 1B). Unlike *Ccny*, which had a relative stable expression pattern, *Ccnyl1* was upregulated from the age of three weeks, and gradually reached a plateau at stages of sexual maturity (Fig 1C), which indicated its role in later stages of spermatogenesis [19]. The expression of *Ccnyl1* was confirmed at the protein levels (Fig 1D). To gain more detailed insight into the expression of *Ccnyl1*, we isolated the germinal cell populations from seminiferous tubules of the testis from sexually matured mice by FACS (S1A Fig) [20]. The mRNA expression results showed that *Ccnyl1* began to be highly expressed in meiotic cells, and gradually increased until the stages with round spermatids. A dramatic decrease of *Ccnyl1* mRNA levels was found in elongating/elongated spermatids (S1B Fig). Both *Ccnyl1* mRNA and protein levels were negligible in spermatogonia (Fig 1E, S1B and S1C Fig). The immunolabeling and immunoblot results showed that CCNYL1 protein was highly expressed on the membrane of meiotic spermatocytes (Fig 1E white arrows, and S1C Fig). Contrary to the mRNA expression levels, CCNYL1 protein was still abundant on the membrane of the elongating/elongated spermatids (Fig 1E, yellow arrows), but was absent from spermatozoa when the residual body was shed (Fig 1E, asterisks). To confirm the subcellular localization of CCNYL1, we isolated the membrane and cytoplasmic proteins, and demonstrated the expression of CCNYL1 on the membrane proteins fraction (Fig 1F). Taken together, these data provide evidence that CCNYL1 might play a critical role in spermatogenesis.

### Fertility Status and Sperm Function in *Ccnyl1*-/- and *Ccny-/-* Mice

**Fig 2:**
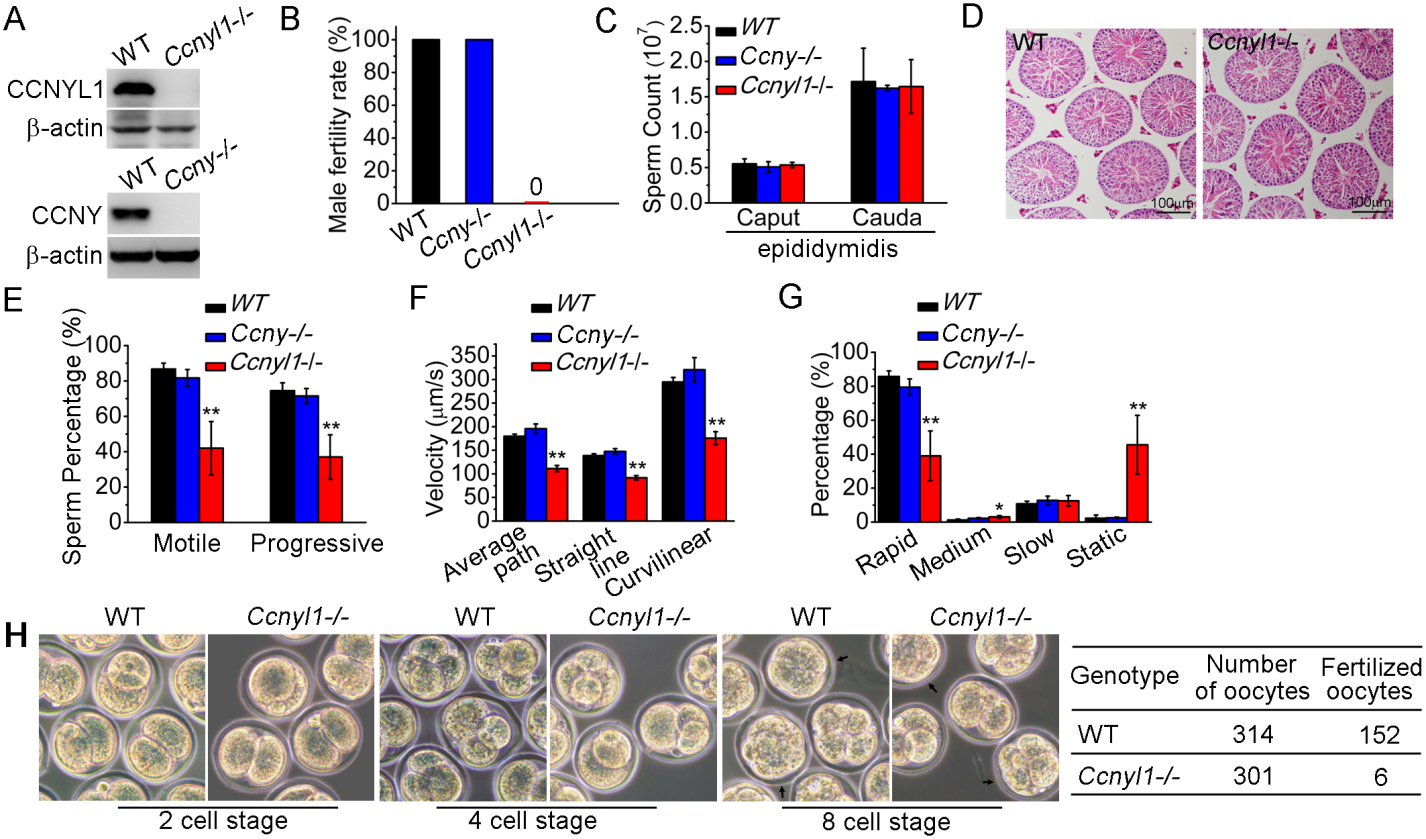
Essential function of CCNYL1 for male fertility and sperm motility. (A) Measurement of CCNYL1 or CCNY protein expression in testes of WT, *Ccnyl1*-/-, and *Ccny*-/- mice, with β-actin serving as loading control. (B) Male fertility of adult WT, *Ccnyl1*-/- and *Ccny*-/- mice (n ≥ 6 per group). Pregnancy was counted in mating cages with male WT, *Ccnyl1*-/-, or *Ccny*-/- mice. (C) Number of spermatozoa in male WT, *Ccnyl1*-/-, and *Ccny*-/- mice collected from the caput and cauda epididymidis (n ≥ 6 per group). (D) HE staining of testicular sections from adult WT and *Ccnyl1*-/- mice. Scale bar: 100μm. (E-G) The percentage of total and progressive motility (E), velocity (F) and the motility distribution (G) of spermatozoa were measured by CASA (n = 5 animals per group). (H) *In vitro* fertilization assay. Oocytes were incubated with cauda epididymidal spermatozoa collected from *WT* or *Ccnyl1-/-* mice. The development of the oocytes was monitored *in vitro*. Data in bar graphs are presented as mean ± SEM. *P < 0.05; **P < 0.01.

To determine the function of *Ccnyl1*, mice with the gene knockout were studied [21] (Fig 2A top panel, S2A Fig). We observed no difference in the phenotype of *Ccnyl1*-/- mice from that of their WT littermates. However, when *Ccnyl1*-/- males were mated with the wild type females, none of the females became pregnant (Fig 2B). In contrast, both male and female *Ccny* knockout mice (S2B Fig) and female *Ccnyl1*-/- mice presented were fertile (Fig 2B and S1 Table), indicating that *Ccnyl1*, but not *Ccny*, was essential for male fertility. Male *Ccnyl1*-/- mice showed normal body weight and reproductive organ weights (S3A and S3B Fig). The serum testosterone and follicle-stimulating hormone levels in *Ccnyl1*-/- mice were not abnormal (S3C and S3D Fig). To elucidate the defects underlying the sterility, we measured the number of spermatozoa obtained from the caput and cauda epididymidis, but observed no difference in the sperm counts between *Ccnyl1*-/- and WT mice in either region (Fig 2C). Furthermore, histological and FACS examination showed no overt effect of *Ccnyl1* deficiency at any stages of germ cell development, which was also supported by similarity in genes specifically expressed by different germinal cell populations (Fig 2D, S3E and S3F Fig). The motility of cauda epididymidal spermatozoa by computer-assisted sperm analysis (CASA) revealed that the percentages of total and progressive motility were greatly reduced in *Ccnyl1*-/- mice (Fig 2E), and the progressive spermatozoa displayed significantly reduced velocity (Fig 2F) though a slightly increased beat frequency (S3G Fig). A higher percentage of spermatozoa from the *Ccnyl1*-/- mice were static or of low motility than that of WT mice (Fig 2G), whereas spermatozoa obtained from *Ccny*-/- mice showed sperm counts and sperm motility no different from those of WT mice (Fig 2C and 2E–2G). Though *Ccnyl1*-/- spermatozoa showed several defects, their capacitation ability was intact (S3H Fig). The ability of *Ccnyl1-/-* mouse spermatozoa to fertilize oocytes *in vitro* was greatly impaired; only six oocytes were fertilized, and two developed to blastocysts *in vitro* compared with WT controls in which 152 oocytes were fertilized and 71 blastocysts obtained in the same set of experiments (Fig 2H). These data indicated that the structural defects and impaired motility of *Ccnyl1-/-* spermatozoa might prevent their propulsion and entry into oocytes, and then lead to impaired fertilization. However, we could not exclude that other uncharacterized defects might also be involved.

Since *Ccnyl1* and *Ccny* share a high similarity in protein sequences, we examined whether *Ccnyl1* and *Ccny* had compensatory effects to each other in mice. As double knockouts of *Ccnyl1* and *Ccny* were embryonically lethal but both *Ccnyl1+/-Ccny-/-* and *Ccnyl1-/-Ccny+/-* mice could be born and grow to adulthood (S4A Fig), these two genes must have a compensatory role in embryonic development. Measurements of the mRNA and protein levels of CCNY in the testis of *Ccnyl1*-/- mice (S4B and S4C Fig), or of CCNYL1 expression in the testis of *Ccny-/-* mice (S4D and S4E Fig) revealed no compensatory expression of these two genes in the testis.

### Morphological Defects of Spermatozoa from CCNYL1 Knockout Mice

**Fig 3:**
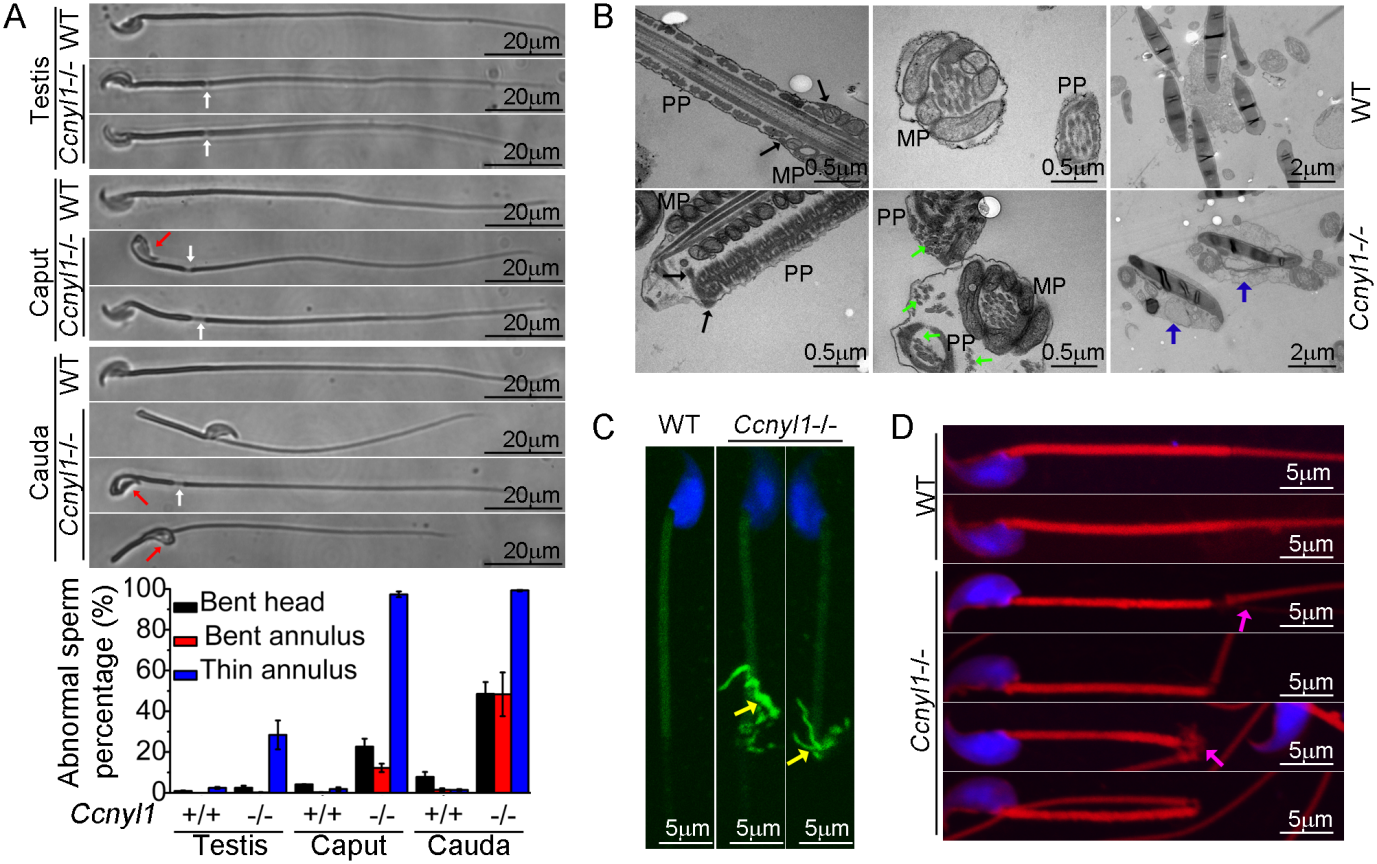
Morphological defects of *Ccnyl1*-/- spermatozoa. (A) Phase Contrast images of spermatozoa collected from the testis, caput and cauda epididymidis of adult WT and *Ccnyl1*-/- mice. *Ccnyl1*-/- spermatozoa showed a thinning of annulus (white arrows) and a bent head wrapped around the neck (red arrows). Scale bar: 20μm. The ratios of defects are summarized in the histograms below (mice: n≥ 3 animals per group). Data are presented as mean ± SEM. (B) TEM images of spermatozoa collected from the cauda epididymidis of adult WT and *Ccnyl1*-/- mice. MP: Middle Piece; PP: Principal Piece. The annulus structure (black arrows) closely linked the MP and PP in WT spermatozoa, but was distant from the MP as the thinning of the annulus region in *Ccnyl1*-/- spermatozoa. Microtubules (green arrows) were dispersed out of the PP in *Ccnyl1*-/- spermatozoa. Two *Ccnyl1-/-* spermatozoa with bent heads wrapped by cytoplasmic contents are shown (blue arrows). Scale bar for the top and middle panels: 0.5 μm, scale bar for the bottom panels: 1 μm. (C and D) Immuno-labeling of α-tubulin (C) and F-actin (D) of spermatozoa collected from cauda epididymidis of adult WT and *Ccnyl1*-/- mice. Microtubules (yellow arrows) and F-actin (pink arrows) were extruded from of this region in spermatozoa of *Ccnyl1*-/- mice, scale bar: 5 μm.

To find the cause of the impaired sperm motility of *Ccnyl1* knockout mice, we examined their sperm morphology. Almost all spermatozoa isolated from the caput and cauda epididymidis of *Ccnyl1*-/- mice displayed thinning of the annulus region (Fig 3A). About half the cauda spermatozoa displayed a sharp bend at the annulus region. Moreover, many sperm head were bent and wrapped around the neck (Fig 3A). In addition, the analysis with differential interference contrast microscope revealed that spermatozoa from the cauda epididymidis of *Ccnyl1*-/- mice lacked of cytoplasmic droplets (S5A Fig). This abnormal frequency was less evident in spermatozoa collected from more proximal epididymal regions (Fig 3A, bottom panel, and S5A Fig). Only a small percentage of *Ccnyl1-/-* spermatozoa showed the morphological phenotype in the testis (Fig 3A). In addition, high expression of CCNYL1 and CDK16 was found in the testicular spermatocytes and spermatids, rather than in the epididymis, and also CDK16 protein levels were no lower in the epididymis of *Ccnyl1-/-* mice than in that of WT mice (S4C and S4G Fig). Moreover, there were no significant changes in epididymal *Ccnyl1* expression in mice of different ages (S4F Fig). Thus, we concluded that it was the lack of *Ccnyl1* in the testis and the subsequently decreased CDK16 protein levels that led to the abnormality of sperms. The gradually increased morphological defects of sperms might be due to the mechanical stress during the transition through the epididymis or the onset of motility of the sperms themselves.

The results of TEM showed that *Ccnyl1*-/- spermatozoa had no mitochondrial sheath at the thinned region, and the annulus structure remained to attach with the dense fiber sheath rather than the mitochondrial sheet (Fig 3B, left bottom panel). Microtubule breakage was also found in *Ccnyl1*-/- spermatozoa, where the microtubules were extruded from the principal piece (Fig 3B, middle bottom panel). This was confirmed by the immunolabeling (Fig 3C). In addition, we observed bent heads and necks that were wrapped around by contents resembling the cytoplasm of *Ccnyl1*-/- spermatozoa (Fig 3B, right bottom panel). Actin cytoskeleton structures were also dispersed out in this region (Fig 3D). The mitochondrial markers cytochrome C and cytochrome C oxidase IV were expressed equally at the protein level between WT and *Ccnyl1*-/- spermatozoa, whereas the β-actin levels were higher in *Ccnyl1*-/- spermatozoa than those of WT mice (S5B Fig). In an attempt to explain the increased β-actin levels in *Ccnyl1*-/- spermatozoa, the key molecules involved in actin polymerization and depolymerization in the testis was measured (S5C Fig), and the F-actin to G-actin ratios in the testis and spermatozoa was compared, but no significant differences from WT were observed (S5D Fig). In addition, we measured the activity of RhoA/Rac1/Cdc42, which is involved in actin cytoskeleton rearrangement [22], but no difference between *Ccnyl1*-/- and WT testes was found (S5E Fig). We also screened the canonical Wnt pathway, which is regulated by the *Ccnyl1* homolog *Ccny*, but found no differences between *Ccnyl1*-/- and WT testes (S5F Fig). These lines of evidence indicate that the increased β-actin levels in *Ccnyl1*-/- spermatozoa might be ascribed to the incomplete shedding of cytoplasmic contents rather than *per se* cytoskeleton defects.

### Screening the Targets for CCNYL1

**Fig 4:**
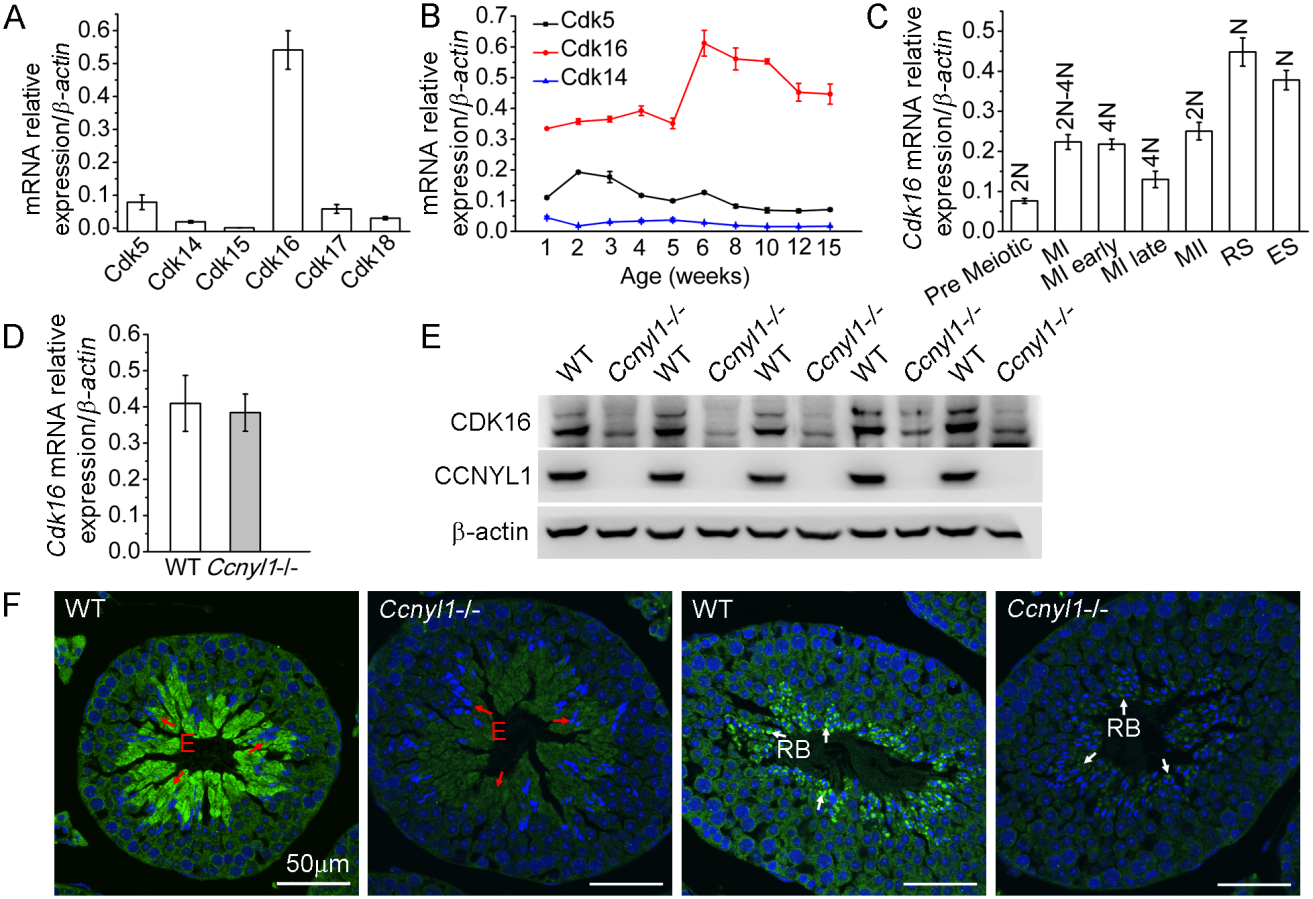
CDK16 protein level is decreased in *Ccnyl1*-/- testis. (A) *Cdks* mRNA levels were measured in testes of adult WT mice (n = 4). Results are presented as mean ± SEM. (B) *Cdk5*, *Cdk14* and *Cdk16* mRNA levels were measured in testes of WT mice at different ages (n = 4). Data are presented as mean ± SEM. (C) *Cdk16* mRNA levels were measured in sorted germ cell populations from adult WT mice. (MI: Meiosis I; MII: Meiosis II; RS: Round Spermatids; ES: Elongating and Elongated Spermatids; N: Haploid; 2N; Diploid; 4N: Tetraploid). Data are presented as mean ± SEM. (D) *Cdk16* mRNA levels were measured in testes of adult WT and *Ccnyl1*-/- mice (n ≥ 4 per group). Data are presented as mean ± SEM. (E) CDK16 protein levels were measured by western blots of testes of adult WT and *Ccnyl1*-/- mice (n = 5 per group). (F) Immunolabeling of CDK16 (green) in testicular sections of two different spermatogenic cycles of adult WT and *Ccnyl1*-/- mice. Nuclei were labeled with DAPI (blue). E: Elongated Spermatids (red arrow); RB: Residual Body (white arrow). Scale bar: 50 μm.

Because cyclins always have CDK partners, we sought testicular CDKs and found that *Cdk16* was highly expressed there (Fig 4A). The expression of *Cdk16* was relatively stable with age (Fig 4B) and was highly expressed in various populations of meiotic germ cells (Fig 4C). The mRNA levels of *Cdk16* in the *Ccnyl1*-/- testis were similar to those of WT mice (Fig 4D). However, the CDK16 protein level was significantly decreased in the *Ccnyl1*-/- testis (Fig 4E). This observation was supported by the immunolabeling results, which showed that the CDK16 protein was most abundant in the elongating/elongated spermatids (Fig 4F and S1C Fig) and even in the residual bodies of WT mice, but the signals were substantially lower in those of *Ccnyl1*-/- mice (Fig 4F). These results suggest that CCNYL1 might interact with CDK16 to regulate spermatogenesis.

### Identification of CDK16 as a Target for CCNYL1

**Fig 5:**
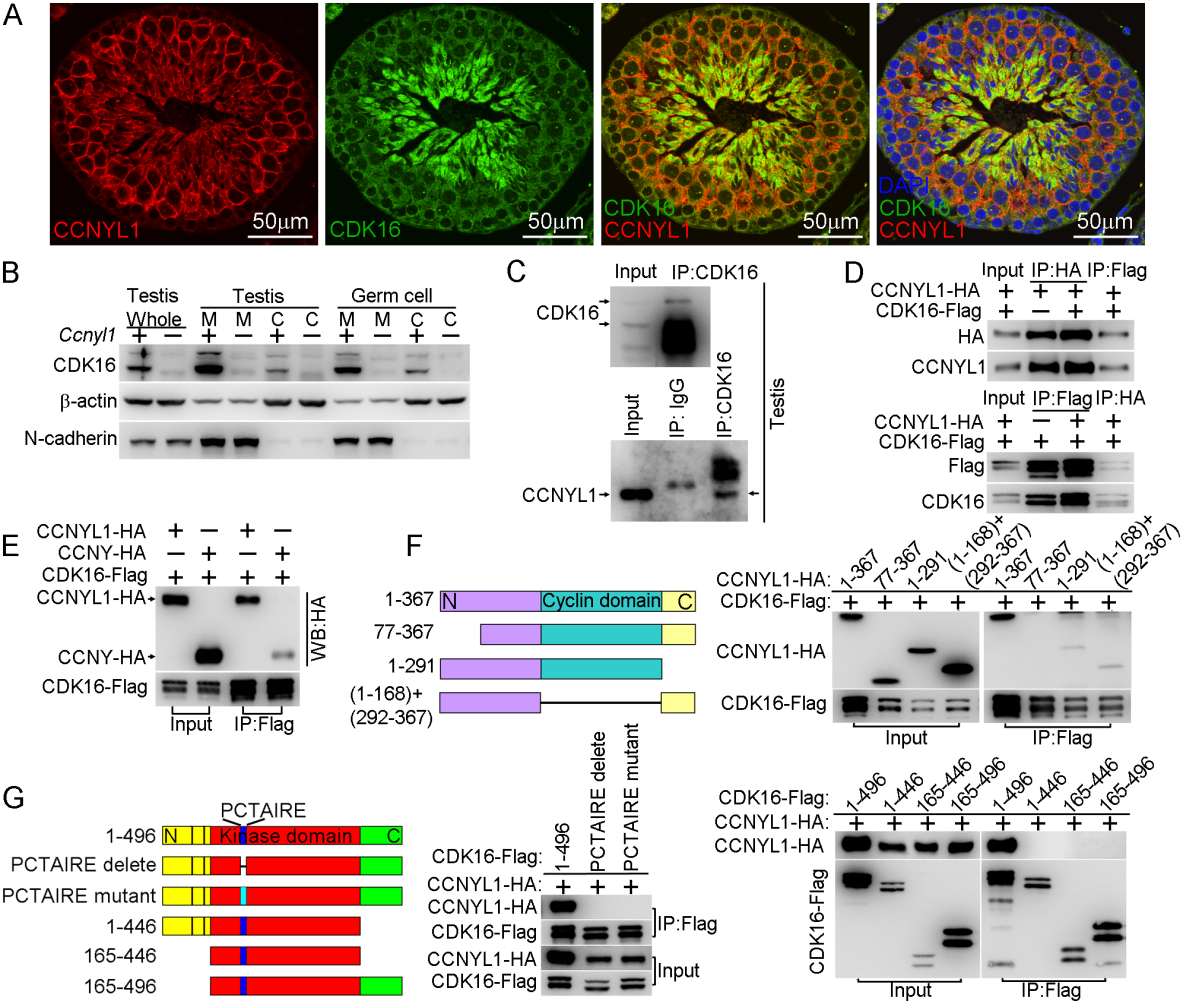
Interaction of CCNYL1 with CDK16. (A) Co-immunolabeling of CCNYL1 (red) and CDK16 (green) in testicular sections of WT mice. Nuclei were labeled with DAPI (blue). Scale bar: 50 μm. (B) CDK16 protein expression was measured in membrane extracts and cytoplasmic extracts isolated from testes or germ cells of WT and *Ccnyl1-/-* mice. N-cadherin served as the membrane protein control, while β-actin served as the cytoplasmic protein control. (C) CDK16 protein was immunoprecipitated from testicular lysate with CDK16 antibody. Both CDK16 and the Co-immunoprecipitated CCNYL1 were analyzed by western blotting. (D) HEK293T cells were co-transfected with N-terminal tagged CCNYL1*-*HA and C-terminal tagged CDK16-Flag plasmids. CCNYL1-HA and CCNYL1-Flag proteins were co-immunoprecipitated using either anti-HA or anti-Flag conjugated agarose before analyzed by western blot. (E) HEK293T cells were co-transfected with CDK16-Flag and CCNYL1-HA, or CDK16-Flag and CCNYL-HA plasmids, proteins were co-immunoprecipitated by using anti-Flag conjugated agarose before analyzed by western blotting. (F) Truncated CCNYL1-HA variants were coexpressed with CDK16-Flag in HEK293T cells. The interactions were analyzed by CoIP experiments followed by western blotting. (G) CDK16-Flag variants were co-expressed with CCNYL1-HA in HEK293T cells. The interactions were analyzed by CoIP experiments followed by western blotting. PCTAIRE is the conserved sequence of CDK16 protein. PCTAIRE mutant (PCTAIRE was mutated to ACAAIAA).

The interaction of CCNYL1 with CDK16, studied in co-immunolabeling experiments, showed that CCNYL1 partially colocalized with CDK16, particularly at the late stages of spermatogenesis (Fig 5A). Unlike CCNYL1, which was specifically localized on the membrane, CDK16 was localized on the membrane and in the cytoplasm (Fig 5B). Further analysis of the interaction between these two proteins, involved coimmunoprecipitation (CoIP) in testicular tissues and in protein over-expressed HEK293T cell lines. These experiments revealed that CCNYL1 specifically interacts with CDK16 (Fig 5C and 5D), which was also confirmed by the coimmunolocalization results in HEK293T cell lines (S6 Fig). We also tested the interactions between CCNY and CDK16; CCNYL1 had a significantly stronger interaction towards CDK16 than did CCNY (Fig 5E). To examine further the interactions of CCNYL1 and CDK16, we generated truncated fragments of CCNYL1 for CoIP. The results showed that deletion of either the N terminal domain, the cyclin domain, or the C terminal domain of CCNYL1 abrogated or greatly impaired the interactions (Fig 5F). On the other hand, deletion or mutation of the PCTAIRE sequence of CDK16, which is known to mediate the binding of other cyclins [10, 23], completely disrupted the interaction. We then investigated which domains in CDK16 were required for the interaction, and found both its N-terminal and C-terminal were essential for CCNYL1 binding, and the expression of the protein kinase domain alone failed for the recruitment of CCNYL1 (Fig 5G).

### Interaction of CCNYL1 and CDK16 and Maintenance of Protein Stability

**Fig 6:**
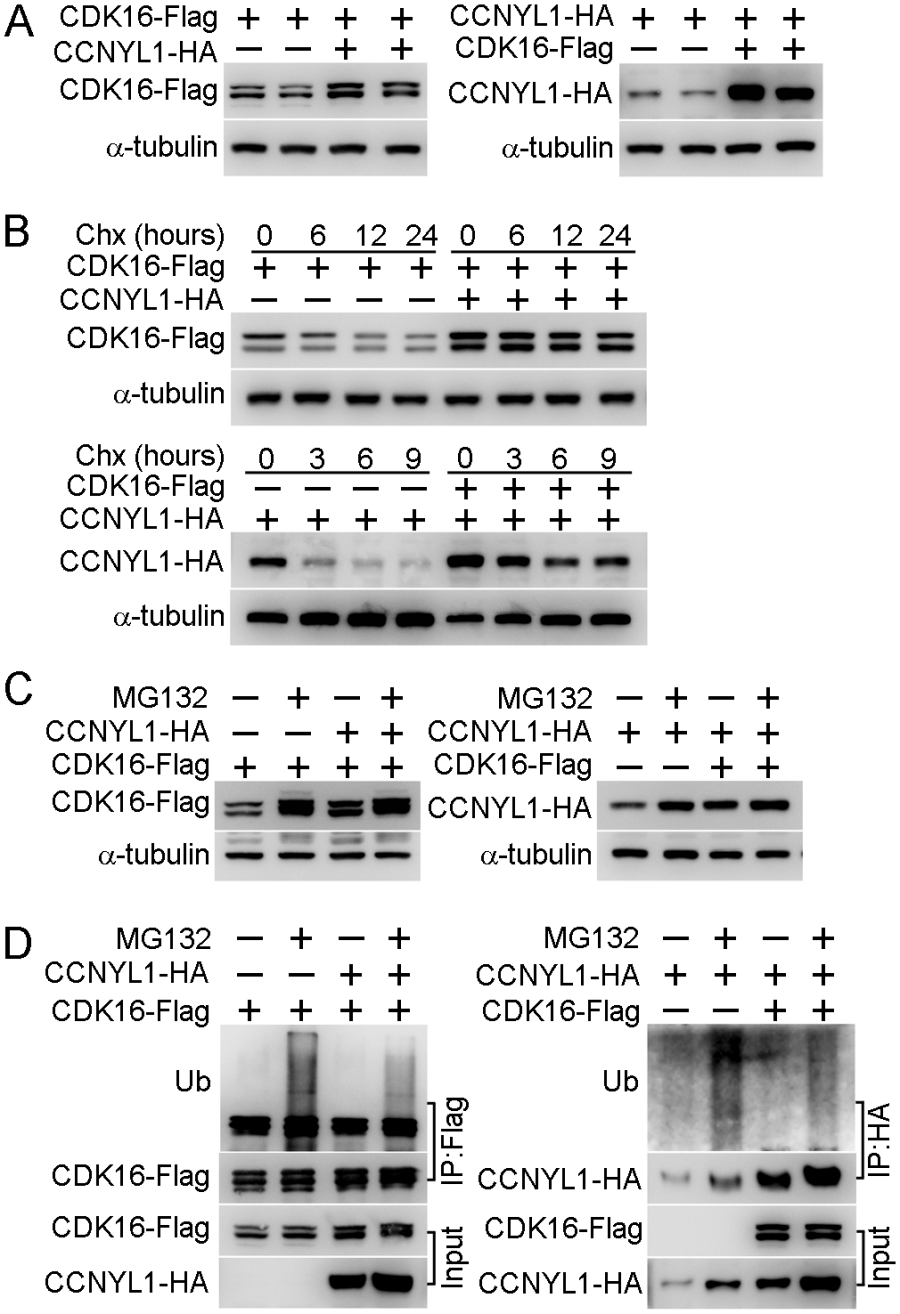
The interaction of CCNYL1 and CDK16 and protection of protein stability. (A) CDK16-Flag and CCNYL1-HA were either expressed alone or together in HEK293T cells. Protein expression levels were measured by western blotting. (B) CDK16-Flag and CCNYL1-HA were either expressed alone or together in HEK293T cells for 24 hours followed by incubation with 10 μM cycloheximide for the indicated times. Protein levels were measured by western blotting. (C) CDK16-Flag and CCNYL1-HA were either expressed alone or together in HEK293T cells for 24 h followed by incubation with 10 μM MG132. Then, cells were harvested at 24 h for the detection of CDK16-Flag, or at 6 h for the detection of CCNYL1-HA. Protein levels were measured by western blotting. (D) CDK16-Flag and CCNYL1-HA were either expressed alone or together in HEK293T cells for 24 hours followed by incubation with 10 μM MG132 for 6 h. The CDK16-Flag and CCNYL1-HA protein were immunoprecipitated for the detection of their ubiquitination levels by western blotting.

Since our data showed that the CDK16 protein level was decreased in the *Ccnyl1*-/- testis (Fig 4E and 4F), and that CCNYL1 interacted with CDK16 both *in vivo* and *in vitro* (Fig 5C and 5D), we proposed that the recruitment of CDK16 by CCNYL1 increases its stability. This hypothesis was supported by the co-expression of CCNYL1 and CDK16 in HEK293T cells resulting in higher levels of both CCNYL1 and CDK16 proteins than single-expression controls (Fig 6A). Protein degradation rates monitored by the cycloheximide chase assay revealed attenuated degradation rates after co-expression (Fig 6B), suggesting that the interaction stabilizes both CCNYL1 and CDK16 proteins. Furthermore, proteasome inhibitor MG132 supplementation led to accumulation of both CCNYL1 and CDK16 proteins, which was more obvious after single expression than co-expression (Fig 6C). Consistent with these results the accumulated ubiquitinated CCNYL1 and CDK16 levels were significantly lower after co-expression than those of after single expression (Fig 6D).

### Importance of Phosphorylation of CDK16 for CCNYL1 Binding and CDK16 Kinase Activity

**Fig 7:**
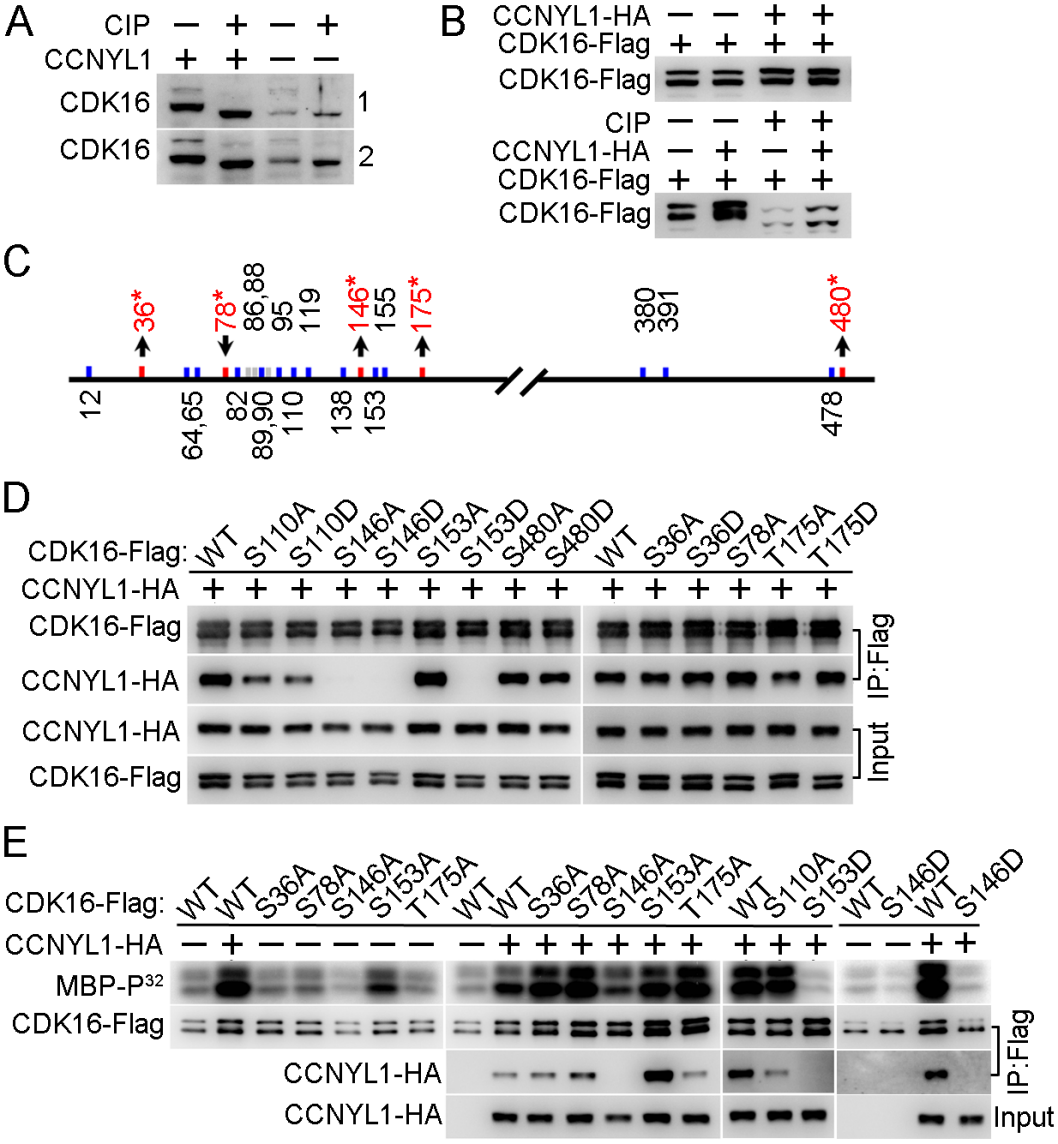
Identification and functional study of phosphorylation modifications of CDK16. (A) Testicular lysates from two pairs of WT and *Ccnyl1*-/- mice were incubated with or without calf intestinal phosphatase (CIP) at 37°C for 30 min and analyzed by western blotting. (B) CDK16-Flag was either expressed alone or together with CCNYL1-HA in HEK293T cells. The cell lysates were incubated with or without CIP, and analyzed by western blotting. (C) Phosphorylation sites on CDK16 identified by mass spectrometry. Sites that changed significantly in phosphorylation levels between single- (CDK16-Flag) and double- (CDK16-Flag and CCNYL1-HA) expressed groups are labeled red with asterisks. (D) Various CDK16-Flag mutants were coexpressed with CCNYL1-HA in HEK293T cells. The interactions were analyzed by CoIP experiments followed by western blot. (E) Varies CDK16-Flag mutants were either expressed alone or coexpressed with CCNYL1-HA in HEK293T cells. The CDK16-Flag protein was immunoprecipitated, and incubated with MBP as substrate in the kinase buffer. Reaction products were separated by SDS-PAGE and followed by autoradiography.

We found that CDK16 was not only decreased in the *Ccnyl1*-/- testis, but also shifted to a slightly lower mass than that of WT controls by SDS-PAGE (Figs 5B and 7A). This band-shift was eliminated by incubating the total testicular cell lysates with phosphatase (Fig 7A), an observation also found in HEK293T cells (Fig 7B), implying that the presence of CCNYL1 is related to the phosphorylation status of CDK16. To check this implication, we co-expressed CCNYL1 with kinase dead CDK16 (K194R). While the interaction between these two proteins was not affected by the kinase dead mutation of CDK16, the slight band-shift of CDK16 was absent (S7A Fig), indicating that the phosphorylation modifications of CDK16 in the presence of CCNYL1 might also depend on the kinase activity of CDK16. Analysis of the phosphorylation sites on CDK16 by mass spectrometry showed that the N-terminal region of CDK16 was highly phosphorylated (Fig 7C). In total, 22 phosphorylation sites were identified, and 19 of them determined with high confidence (S2 Table and S1 Dataset). Of those 19 sites, nine have not been reported. They were S36, S64, S65, S89, S146, T175, T380, S391, and S478. Our results showed that the phosphorylation levels of CDK16 were increased at four sites (S36, S146, T175, and S480) and decreased at one site (S78), when co-expressed with CCNYL1 (S2 Table), consistent with the band-shift of CDK16 reflecting altered phosphorylation levels. Phosphorylation of CDKs has been reported to be involved in modifying their interaction with the cyclins and activation of their own Ser/Thr protein kinase activity [5, 15, 24]. To determine if the altered phosphorylation of CDK16 can affect its binding activity, we generated mutations of these phosphorylation sites for use in CoIP assays. We found that the S36A, S36D, S480A, and S480D mutations had no effect on CCNYL1 binding; but the S78A and T175D mutations increased and the T175A mutation decreased binding activity (Fig 7D). Most significant was the S146 site, with both S146A and S146D mutation almost entirely abolishing the binding to CCNYL1, confirming the importance of this site for the binding activity. In addition, we found S110, S119 and S153 were also highly phosphorylated, of these sites S119 and S153 are reported to be critical for the binding of the CCNY [5]. We mutated the S110, S119 and S153 sites, and found that the S110A, S110D, S119A and S153D mutations decreased the binding towards CCNYL1 to different degrees, whereas the S153A mutation increased the binding to CCNYL1 (Fig 7D and S7B Fig).

In addition, we investigated the kinase activity of CDK16, by a radio-label assay, and showed that the binding of CCNYL1 markedly increased the kinase activity of CDK16 (Fig 7E and S7A Fig). We also explored the phosphorylation modifications on CDK16 kinase activity. Here, we found that the S146A and S146D mutations significantly decreased the kinase activity of CDK16. Co-expression of various CDK16 mutants with CCNYL1 all increased their kinase activity compared with that after single expression. However, S146A, S146D and S153D mutations still displayed substantially lower kinase activity than that of other mutants while co-expressed with CCNYL1 (Fig 7E), demonstrating the importance of these sites in modulating CDK16 kinase activity.

## Discussion

Spermatogenesis involves both mitosis and meiosis processes and produces millions of spermatozoa daily. Understanding the intricate mechanisms that govern germ cell development has important implications for human health and reproduction. The regulation of the cell cycle during production and differentiation of germ cells is a rigidly-controlled process, and close co-operation between specific sets of cyclin:CDK partnerships is necessary for ensuring orderly progression through the cycle [11, 25–26]. It is known that different cyclins have distinct patterns of expression in male germ cells, which suggests different roles for these cyclins in spermatogenesis [25–26]. For instance, *Cyclin A1* is expressed in late pachytene to diplotene spermatocytes, and is required for entering of metaphase I in mouse spermatocytes [27]. However, the precise roles of many cyclins have not been well studied in spermatogenesis, mutant, knockout and conditional knockout mouse models have become valuable tools for exploring cyclin functions. It has been reported that conditional knockout of *Cdk16* in mice leads to male infertility; CDK16 protein interacts with CCNY protein [5], and CCNY is highly expressed in the testis in comparison with its levels in the brain and heart [28]. However, our study showed that the *Ccny*-/- male mice were reproductively normal, but that genetic deletion of *Ccnyl1* leads to infertility of male, but not female, by cooperating with CDK16 and maintaining the enzymatic activity of the CCNYL1:CDK16 complex at the plasma membrane of spermatogenic cells.

Unlike its homologue, *Ccny*, which had a relatively uniform expression pattern, *Ccnyl1* was specifically expressed at high levels in the testis. Given that *Ccnyl1* and *Ccny* homologues have high similarity, we postulated that *Ccnyl1* and *Ccny* were functionally redundant. However, our study showed that *Ccnyl1* and *Ccny* had no compensatory expression in adult testis or epididymis. Additionally, CDK16 protein levels were decreased in the testis of *Ccnyl1-/-* mice, but not in the testis of *Ccny-/-* mice (S4C and S4E Fig). Thus, *Ccnyl1* and *Ccny* might have specialized and different roles in the testis and epididymis. In the thymus, the expression of CCNYL1 was increased in *Ccny-/-* mice, suggesting that *Ccnyl1* has a compensatory role of *Ccny* in this organ. Furthermore, the gene expression profiles showed that *Ccny* had obviously higher expression than *Ccnyl1* in most organs, which may imply a more general functional role of *Ccny*. More interestingly, we found that *Ccnyl1-/-Ccny-/-* mice were embryonically lethal, which strongly suggests a critical role of *Ccnyl1* and *Ccny* in embryonic development, and makes the underlying mechanisms worthy of future investigations. Since the various cyclin members expressed in the testis may have specialized roles in different development stages and cell types, as well as in different cellular activities, for germ cell production and differentiation [25–26]. We therefore postulate that while some cyclins are redundant on certain occasions, they might have different functions on other occasions. The spatial and temporal expression patterns of these genes may provide clues for their respective functions.

To elucidate the mechanism action of CCNYL1, we screened the targets of CCNYL1 by cDNA microarray analysis (accession no. GSE67391). But CCNYL1 showed no obvious roles in transcriptional regulation. *Septin4*-/- spermatozoa showed a similar phenotype with both a thinning annulus and a bent head, but also showed defects in mitochondrial and capacitation function [29–30]. *Sepp1*-/- and *Tat1*-/- spermatozoa displayed a thin annulus and also mitochondrial or capacitation defects [31–32], whereas *Spem1*-/- spermatozoa showed a membrane-enwrapped head, but no annulus defects [33]. Interestingly, spermatozoa collected from *Cdk16*-/- mice displayed a quite similar phenotype to *Ccnyl1-/-* sperm cells [5]. However, that paper reported that CCNYL1 had no interactions with CDK16 from the use of *in vitro* cell line system expressing YFP-tag CCNYL1, which conflicts with our results. As both the N- and C-terminal regions of CCNYL1 are indispensable for the binding of CDK16 to form the functional CCNYL1:CDK16 complex, as shown in the present study, we may explain the conflicting report by the large YFP-tag of YFP-CCNYL1, which may hinder the interactions between CCNYL1 and CDK16.

Since the members of the cyclin family always have CDK partners, we screened the CDKs candidates of interest and found the CDK16 protein level to be decreased in the *Ccnyl1*-/- testis. Co-immunolabeling and CoIP experiments proved that CCNYL1 interacted with CDK16, so that CCNYL1 is no longer an “orphan” cyclin. Moreover, CCNY has been reported to interact with CDK16 [5], which was confirmed in our study. However, the interaction between CCNY and CDK16 was obviously weaker than that of CCNYL1 and CDK16. Thus, CCNY may be a more appropriate partner for other CDKs, or may play a different role other than spermatogenesis and fertility, while CCNYL1 is essential for spermatogenesis by cooperating with CDK16. While CCNYL1 is predicted to have a molecular mass about 42kDa, but it actually showed a band around 53kDa, which indicates the existence of post-transcriptional modification on CDK16. Unlike for CDK16, phosphorylation seems not to be the key factor responsible for this, as phosphatase treatment only caused a small shift of CCNYL1. The positions of truncated CCNYL1 bands indicated that the modification occurred among the first 76 amino acids of the N-terminal region (Fig 5F), which is probably critical for the binding of CDK16 and the localization of CCNYL1.

Further studies showed that the interaction of the proteins CCNYL1 and CDK16 mutually increased their stability, and that ubiquitin/proteasome pathways were involved. While microRNA might target the *Cdk16* mRNA in the *Ccnyl1-/-* testis, it is unlikely to be the key factor decreasing CDK16 protein in the testis of *Ccnyl1-/-* mice. First, there was no difference in testicular *Cdk16* mRNA levels of WT and *Ccnyl1-/-* mice. Secondly, faster CDK16 protein accumulation was observed in HEK293T cells transfected with CDK16 alone than in those transfected with CCNYL1 and CDK16 after MG132 blocking of protein degradation. Thus, the significantly decreased CDK16 protein levels in the *Ccnyl1*-/- testis could be explained by the decreased protein stability as a result of the lack of CCNYL1. We speculate that the interaction of CCNYL1 and CDK16 probably changes the structure or special layout of both proteins, and consequently impairs the ubiquitination process required for the proteasome degradation. As found for other members of the CDKs, CDK16 requires binding of the regulatory partner, CCNYL1, to display its kinase activity. Previous investigations have demonstrated that phosphorylation of CDKs is essential for the binding of cyclin partners and activating their own protein kinase activity [5, 15, 24]. Here we demonstrated that the phosphorylation of N-terminal of CDK16 was not only critical for the binding of CCNYL1, but also essential for the regulation of CDK16 kinase activity. Of these sites, the S146 and S153 were more critical for the binding of CCNYL1 and for modulating CDK16 kinase activity. All these findings are helpful in understanding the regulation and function of CDK16.

Our study showed that CCNYL1 interacts with CDK16 maintaining its stability and kinase activity. This would explain the early expression of CCNYL1 in meiotic spermatocytes, if it were required for the accumulation of CDK16 protein in later stages. Cyclins are known as more than the enzymatic activators of CDKs, and the subcellular localization of many cyclins are well suited to their functions [34]. Since the binding of CCNYL1 strikingly increased CDK16 kinase activity, the localization of CCNYL1 and CDK16 provides evidence that CDK16 could be recruited to the membrane for the access to its substrates, which are still unidentified yet. Our study showed that both CCNYL1 and CDK16 were highly expressed at late stage of spermatogenesis, from which we deduced that CCNYL1 and CDK16 partners play an important role in the terminal stage of spermatogenesis.

Although spermatogenesis has been widely studied, the molecular and cellular mechanisms underlying the late stages of spermatogenesis, such as the removal of residual bodies and the formation of the annulus, remain largely unknown [35, 36]. Given that PCTAIRE protein kinases interact directly with the COPII complex in modulating secretory cargo transport [18], and PCTAIRE1 phosphorylates *N*-ethylmaleimide-sensitive fusion protein, which regulates exocytosis [17], vesicle or membrane trafficking dysfunction may be responsible for the *Ccnyl1*-/- sperm defects. Although no obvious abnormality of the vesicles from *Ccnyl1*-/- spermatids was observed through TEM analysis of the testicular tissue, *Ccnyl1*-/- spermatids were abnormal in shedding residual bodies (Fig 3B, right panel). Overall, more investigations are needed for the identification of the substrates of CCNYL1:CDK16 complex and their role in the late stages of spermatogenesis; they should focus on the plasma membrane of spermatocytes. Additionally, the binding efficiency of two CDK16 antibodies used here for immunoprecipitation was low, so development of antibodies with high binding efficiency is essential for identifying the targets of CDK16 by mass spectrometry analysis. It is notable that spermatozoa with genetic deletions of *Sept4* [29–30] or *Ccnyl1* (this study) share the common structural defects of an annulus detached from the posterior segment of spermatozoa. The defective spermatozoa also show variations in other functions, which may be attributable to the function of different genes in the annulus-related biological processes. As it is known that the annulus appears as an electron-dense structure assembled in the cytoplasm at very early stages of germ cell differentiation [37], when CCNYL1 and CDK16 proteins are highly expressed and co-localized, we hypothesize that the CCNYL1:CDK16 complex may be involved in the shedding of the residual body and membrane separation *via* a septin-dependent pathway. Consistent with this hypothesis, CDKs regulate septins organization and phosphorylation in yeasts [38–40]. However, the mechanisms need further study.

In summary, we conclude that the CCNYL1 and CDK16 protein complex is essential for spermatogenesis and male fertility in the mouse, and that, as CCNYL1 is specifically expressed in the testis, and *Ccnyl1*-/- mice, although sterile, appeared healthy, with normal testosterone levels and mating ability, CCNYL1 may be an ideal molecule for a male contraceptive, or for screening or development of drugs that specifically inhibit CCNYL1 expression or CCNYL1/CDK16 activity; this could be very promising for the translational research. Finally, research from mouse models continues to enrich our knowledge of the genetic basis of male infertility. These studies should be beneficial for translating knowledge from animal models into strategies for diagnosis and treatment of human male infertility.

## Materials and Methods

### Animals

*Ccnyl1* ^tm1a (EUCOMM) Wtsi^ mice were obtained from the European Conditional Mouse Mutagenesis Program. The strain was targeted KO-first, and here we designated the strain as *Ccnyl1* knockout [21]. Genotypes were identified by PCR analysis (F: 5’- GAAAAGAGGAAGAGCAACCATTTA-3’ R: 5’- ATACACTCTTGCAGGCATACACAT-3’). The wild type allele generated a band at 350bp, while the KO-first allele was at 400bp. The KO-first allele was confirmed by Lacz specific primers (200bp) (Lacz-F: 5’- CGACCCGCATTGACCCTA -3’, Lacz-R: 5’- TCGCCATTTGACCACTACCA -3’), see also in S2A Fig. Since male *Ccnyl1*-/- mice were sterile, these mice were produced using heterozygous pairings. *Ccny* knockout mice were obtained from the Shanghai Research Center for Model Organisms. The mice obtained were mixed backgrounds of 129 and C57BL/6J, and were back-crossed to C57BL/6J for five generations for the subsequent studies. The *Ccny* knockout mice were conditional knockout mice, which were obtained by crossing the *Ccny* loxP-flanked mice to a strain carrying the EII-Cre to generate founder mice with the *Ccny* deletion in germinal cells, from which the offspring with *Ccny* knockout of the entire body were generated. *Ccny*-/- mice genotypes were identified by PCR analysis (F1: 5’-AATACAGCTCTTGCTCCACCA-3’, F2: 5’-GCTACCCGTGATATTGCTGAA-3’, and R1: 5’ CCTAGCCTCCAAGAGCACATA-3’). The wild type allele generated a single band at 450bp, while the KO allele generated a single band at 750bp, see also S2B Fig. All animals were maintained in strict accordance with the guidelines of the Institutional Animal Care and Use Committee of the Institute of Biochemistry and Cell Biology, Shanghai, China. All animals were housed in standard specific pathogen free animal house, kept on a 12-hour light/dark cycle, and were given access to food and water *ad libitum*. All experimental protocols and animal experiments were approved by the Institutional Animal Care and Use Committee of the Institute of Biochemistry and Cell Biology, Shanghai Institutes for Biological Sciences, and Chinese Academy of Sciences (Permit Number: SIBCB-NAF-14-001-s304-007, and SIBCBSPF0034). All efforts were made to follow the Replacement, Refinement and Reduction guidelines. To minimizing suffering, mice were anaesthetized with sodium pentobarbital before being killed.

### RNA Extraction, qRT-PCR, Plasmids and Mutagenesis

Total RNA was isolated from tissues or cells by using Trizol (Invitrogen). The RNA was reverse-transcribed with M-MLV reverse transcriptase (Takara). qRT-PCR was performed using a Real-Time System (Toyobo). Raw data were normalized to the internal control and presented as a relative expression level. All primers for qRT-PCR are described in S3 Table. *Cdk16*, *Ccnyl1*, and *Ccny* sequences were amplified from the testicular cDNA from WT mice and cloned into PCMV-3tag3a and PCDNA3.0 (reconstructed with HA tag at the N-terminal) separately. Mutagenesis was carried out by using QuikChange Lightning Site-Directed Mutagenesis Kit (Agilent). All coding sequences were confirmed by DNA sequencing.

### Sperm Preparation, Motility Analysis, Capacitation Assay, Measurement of Testosterone and Follicle-Stimulating Hormone

Enriched Krebs-Ringer bicarbonate medium (without CaCl_2_, without NaHCO_3_, without BSA) was used throughout the study for mouse sperm preparation [41]. The sperm suspension was either taken for computer-assisted sperm analysis (CASA) or for other assays. For the capacitation assay, spermatozoa from the cauda epididymidis were incubated in the EKRB medium (with CaCl_2_, NaHCO_3_, and BSA) for 60 min and 90 min. Phosphotyrosine levels were analyzed by western blotting. Testosterone and follicle-stimulating hormone levels in mouse serum were measured by enzyme-linked immunosorbent assay (R&D, Jiancheng Bioengineering Nanjing) according to the manufacturer’s instructions.

### Cell Transfection, Cell Treatment, Co-immunoprecipitation (Co-IP), In Vitro Kinase and De-phosphorylation Assays

Cell transfection was performed with Lipofectamin 2000 (Invitrogen). For stability analysis, transfected cells were treated with 10 μM cycloheximide (Sigma) or 10 μM MG132 (Selleck) for various times as indicated in the results section. For IP and CoIP assay, cells or tissues were lysed in IP buffer (150mM NaCl, 50mM Tris, 1mM EGTA, 0.2% (v/v) NP-40, Sigma Protease Inhibitor Cocktail, Selleck Phosphatase Inhibitor Cocktail). The supernatant was incubated with monoclonal anti-HA/Flag-conjugated agarose (Abmart) or CDK16 antibody (1:40) at 4°C for 2 hours. The beads were washed with IP buffer three times, and proteins were eluted with glycine and subjected to western blot analysis. For kinase assay, HEK293T cells were transfected with CDK16-Flag (WT or mutants) together with CCNYL1-HA. CDK16-Flag was immunoprecipitated by anti-Flag agarose and washed three times with IP buffer and twice with kinase buffer (50 mM Hepes, 10 mM MgCl_2_, 0.1 mM Na_3_VO_4_, and 0.5 mM DTT). The kinase activity was measured at 30°C for 1 hour after addition of 10 μg MBP substrate (Sigma), 5 μCi γ˗P^32^-ATP (PerkinElmer), and 20 μM ATP (Takara) in 50 μl reaction system. The reaction products were separated by SDS-PAGE and followed by radioautography. For the dephosphorylation assay, testis or transfected cells were lysed in a Tris buffer (50 mM Tris-HCl, pH 8.0, 150 mM NaCl, 2 mM EDTA, 0.5 mM DTT, 0.5% (v/v) Triton X-100, with protease inhibitor cocktail (Sigma). 80 μg lysate was incubated in the presence or absence of calf intestine phosphatase (CIP, 20 units, New England Biolab) at 37°C for 30 min and analyzed by western blotting.

### Western Blotting, Immunofluorescence and Transmission Electron Microscopy Analyses

Samples of cells or tissues were separated on SDS-PAGE gels, transferred to nitrocellulose membranes and probed with primary antibodies as follows: CCNYL1 (provided by Professor Xueliang Zhu, Shanghai Institute of Biochemistry and Cell Biology); CCNY (Abcam); α-tubulin, β-actin (Sigma); N-cadherin (BD); CDK16 (Santa Cruz, sc-53410, SC-174), Ubiquitin, HA, Flag, Cofillin, Cofillin1 Ser3, Profilin-1, Profilin-2 (Santa cruz); β-catenine, β-catenine Ser45/Thr41, β-catenine Ser33/37/Thr41, Gsk-3β, Gsk-3β-Ser9 (Cell Signaling Technology); Phosphotyrosine (Millipore), and followed by secondary antibodies conjugated to horseradish peroxidase at a dilution of 1:5000 (Santa cruz) and detection by the ECL System (Pierce). For immunohistochemistry, mouse tissues were fixed in 4% paraformaldehyde and embedded in paraffin. Sections (5 μm) were deparaffinized, perforated, blocked (3% BSA) and then incubated with CCNYL1 and/or CDK16 primary antibody, followed by incubation for 1 hour with FITC-conjugated goat-anti-mouse and/or Cy3-conjugated donkey-anti-rabbit secondary antibody (Molecular Probes) at room temperature, then co-labeled with DAPI (Sigma). The localization of F-actin of spermatozoa was revealed by using TRITC-conjugated phalloidin (1 μg/ml, Sigma) for 30 min at room temperature. Sperm microtubules were revealed by incubation with α-tubulin primary antibody followed by FITC-conjugated secondary antibody. Photomicrographs were taken with an Olympus FV1200 confocal microscope. Phase-contrast images were acquired using a Zeiss Axioskop 2 microscope. DIC images were acquired using an Olympus BX51 system microscope. For Transmission Electron Microscopy, spermatozoa collected from cauda epididymidis were fixed with 2.5% glutaraldehyde at 4°C overnight. After a few washes with 0.1M phosphate buffer, the spermatozoa were fixed with 2% osmic acid, washed and dehydrated through an ethanol series and embedded in Epon812 resin. Ultrathin sectionings (70 nm) were prepared on an EM UC6 ultramicrotome (Leica). Sections were labeled with 1% lead citrate for 5 min and 2% uranyl acetate. Images were acquired from a Tecnai Spirit 120 kV transmission electron microscope (FEI).

### Isolation of Spermatogenic Cells, Flow Cytometric Analysis and Cell Sorting, Extraction of Membrane and Cytoplasmic Protein, Isolation of F-actin/G-actin, and Measurement of Cdc42/Rac1/RhoA Activity

In brief, a three-step enzymatic digestion method was used. The seminiferous tubules were digested twice with collagenase type I (Worthington) at 200U/ml for 15 min at 33°C in DMEM (Gibco) supplemented with DNase I (10U/ml, Promega). The pellets were further digested with 0.05%Trypsin-EDTA (Gibco) for 5 min and stopped by 2% serum. The resulting cell suspension was filtered through a 40 μm cell strainer (BD). The harvested cells were diluted for Hoechst (5 μg/ml) and PI (2 μg/ml) staining, and then prepared for FACS analysis (LSR II Flow Cytometer, BD) or cell sorting (Aria II Flow Cytometer, BD). Membrane and cytoplasmic proteins were extracted with a membrane and cytoplasmic protein extraction kit (Beyotime Biotechnology). The F-actin/G-actin was isolated by the In Vivo Assay Biochem Kit (Cytoskeleton). The RhoA/Rac1/Cdc42 activities were measured by G-LISA Activation Assay Kit (Cytoskeleton).

### *In Vitro* Fertilization Assay

C57BL/6J females were injected i.p. with 2.5 U PMSG followed by 5U hCG 48 h later. Oocytes were collected 13 h after the injection of hCG. Spermatozoa were capacitated for 1.5 hours and mixed with the oocytes in 500 μl of HTF fertilization medium, and incubated for 6 hours at 37°C in 5% CO_2_, 5% O_2_ and 90% N_2_. Eggs were washed and transferred to HTF medium covered with mineral oil (Sigma). The fertilized and unfertilized oocytes were counted 48 h later. They were then washed and transferred to KSOM medium for culture to blastocyst stage. Images were taken using Olympus IX71 microscopy.

### cDNA Microarray Analysis

Testicular tissues were quickly obtained from two month old *Ccnyl1-/-* and WT mice, frozen in liquid nitrogen. After thawing, tissues were then placed into the Trizol solution (GibcoBRL) for extraction of total RNA according to the manufacturer's instructions. Biotinylated cRNA samples were prepared according to the standard Affymetrix protocol from 6 μg total RNA per each sample. Following fragmentation, 15 μg cRNA of each sample was hybridized for 16 hours at 45°C on GeneChip Mouse Genome Array. Then GeneChips were washed and stained in the Affymetrix Fluidics Station 450, and scanned in the Gene Array Scanner3000. The data were analyzed with Microarray Suite version 5.0 (MAS 5.0) by using Affymetrix default analysis settings and global scaling as the normalization method. The trimmed mean target intensity of each array was arbitrarily set to 100.

### LC-MS/MS Analysis

For mass spectrometric analysis, HEK293T cells were transfected with CDK16-Flag and/or CCNYL1-HA plasmids. Proteins were immunoprecipitated with anti-Flag-conjugated agarose. The eluted proteins were mixed with 5 volumes of urea (8M) and prepared for LC-MS/MS analysis. Two biological replicates were analyzed in three or five technical replicates. All samples were separated by the Easy nLC 1000 system (ThermoFisher Scientific) coupled to the Orbitrap Fusion Tribrid (ThermoFisher Scientific) mass spectrometer. Peptides were separated on a 15 cm column (i.d. 75 μm) packed in-house with the reverse-phase (RP) materials ReproSil-Pur C18-AQ, 3.0 μm resin (Dr. Maisch GmbH, Germany), employing a water/acetonitrile/0.1% (v/v) formic acid gradient from 5–23% (v/v) over 50 min. The Orbitrap Fusion Tribrid was operated in ‘high-high’ mode, in which during a maximum 2 sec cycle, the most abundant multiply-charged parent ions were selected with high resolution (120 000 @ m/z 200) from the full scan (300–1500 m/z) for HCD fragmentation. Precursor ions with singly charged and charge states over 8 were excluded. Resolution for MS2 spectra was set to 15 000 @ m/z 200, target value was 2E6 (AGC control enabled with maximum injection time 100 ms), isolation window was set to 2 m/z and 30.0% NCE. Data were acquired with Xcalibur software. All mass spectrometric data were analyzed using MaxQuant 1.5.1.0 against the mouse [42] uniprot.20130724.fasta database. Carbamidomethyl cysteine was sought as a fixed modification, and oxidized methionine, protein N-terminal acetylation, as well as phosphorylation of serine, threonine, and tyrosine as variable modifications. Enzyme specificity was set to trypsin/P. Two missing cleavage sites were allowed. For MS and MS/MS, the tolerances of the main search for peptides were set at 7 ppm and 20 ppm, respectively. The peptide, protein and site false discovery rate (FDR) was fixed at a significant level not greater than 0.01. The high-confidence phosphorylation sites (class I sites) were used for further analyses, which were selected on the basis of (1) the minimum score for modified peptides is of 40, and (2) the PTM scores (localization probability > 0.75 and probability localization score difference ≥ 5) as described before [43].

### Data Availability

Raw sequencing reads are available from the National Center for Biotechnology Information’s GEO database (www.ncbi.nlm.nih.gov/geo/) under accession no. GSE67391.

### Statistical Analysis

Data are presented as mean ± SEM. All experiments were performed at least in triplicate. Student’s t-test was used for comparison between two groups. *P* < 0.05 was considered significant.

## Supporting Information

S1 Fig Characterization of sorted germinal cells from the testis. (A) Mouse germinal cells were isolated by the FACS sorting method. Propidium iodide (PI) staining was used to exclude the dead cells, while Hoechst 33342 was used to assign the cells into different populations according to their DNA content. (MI: Meiosis I; MII: Meiosis II; RS: Round Spermatids; ES: Elongating and Elongated Spermatids; N: Haploid; 2N; Diploid; 4N: Tetraploid). The fluorescence images were taken just after sorting (Olympus IX71 microscopy). (B) *Ccnyl1* mRNA levels were measured in sorted germinal cell populations from a WT testis. (MI: Meiosis I; MII: Meiosis II; RS: Round Spermatids; ES: Elongating and Elongated Spermatids; N: Haploid; 2N; Diploid; 4N: Tetraploid). The cell populations are also indicated. Data are presented as mean ± SEM. (C) CCNYL1 and CDK16 protein levels were measured in sorted germinal cells by western blotting.
(TIF)

S2 Fig Targeted disruption of *Ccnyl1* and *Ccny*. (A) Structure of targeted *Ccnyl1* locus. The black arrow depicts the primers for genotyping. *Ccnyl1* knockout mice were first knockout with stop codon and polyA tails following LacZ sequences. The gray boxes depict the *Ccnyl1* exons. The neomycin resistance cassette is shown as a light blue box, the loxP sites are shown as red triangles, and the FRT sites are shown as green triangles. (B) Structure of targeted *Ccny* locus. The black arrow depicts the primers for genotyping, and the exons of *Ccny* are shown as gray boxes.
(TIF)

S3 Fig Characteristics of male *Ccnyl1*-/- mice. (A-D) Body weight (A), reproductive organ weight (B), serum testosterone (C) and follicle-stimulating hormone (D) of male WT and *Ccnyl1*-/- mice (n ≥ 5 per group). Data are presented as mean ± SEM for bar graphs and hereafter. (E) Measurement of genes specifically expressed in different germ cell populations of WT and *Ccnyl1*-/- mice (n = 5 per group). Gene expression level was normalized to WT mice, which was defined as 1.0. (F) FACS analysis of the germ cell populations between adult WT and *Ccnyl1*-/- mice. (G) Beat cross frequency of spermatozoa collected from the cauda epididymidis of WT and *Ccnyl1*-/- mice (n = 5 per group). *P < 0.05; **P < 0.01. (H) Spermatozoa collected from the cauda epididymidis of WT and *Ccnyl1*-/- mice were incubated in full EKRB buffer (with Ca^2+^, BSA, NaHCO_3_) for capacitation. Spermatozoa were collected at 0, 60, and 180 minutes after incubation (n = 2 per group). Tyrosine phosphorylation levels were measured by western blotting, with α-tubulin serving as loading control.
(TIF)

S4 Fig Determination of the compensatory effects between CCNYL1 and CCNY. (A) Male and female *Ccnyl1+/-Ccny+/-* mice were intercrossed, numbers of offspring with different genotypes were counted (total: 92 mice). The red numbers are the theoretical distribution according to Mendel's laws of inheritance. (B-C) Both mRNA and protein levels of CCNY expressions were measured in the testis, brain, and caput/cauda epididymidis of adult *Ccnyl1-/-* and WT mice (mRNA: n = 5/group, protein: n = 2-3/group). (D-E) Both mRNA and protein levels of CCNYL1 expressions were measured in the testis, brain, and caput/cauda epididymidis of adult *Ccny-/-* and WT mice (mRNA: n = 5/group, protein: n = 2-3/group). (F) mRNA levels of *Ccnyl1* expression were measured in whole epididymides and white adipose tissues of mice at different ages (n = 4). (G) Immunolabeling of CCNYL1 (red) in sections of the epididymis. Sections from *Ccnyl1-/-* epididymis were stained as the negative control. Nuclei were labeled with DAPI (blue).
(TIF)

S5 Fig Deeper characterization of the abnormality of *Ccnyl1*-/- mice. (A) DIC images of spermatozoa collected from caput and cauda epididymidis of adult WT and *Ccnyl1*-/- mice. Black arrow: cytoplasmic droplets, Scale bar: 25 μm. (B) Measurement of β-actin, Cyc (Cytochrome C) and Cox IV (Cytochrome c Oxidase Subunit IV) protein levels of WT and *Ccnyl1*-/- spermatozoa (n = 3 per group), with α-tubulin serving as loading control. (C) Measurement of Cofilin, p-ser3-Cofilin1, Profilin1, Profilin-2 and β-actin protein levels in testis of WT and *Ccnyl1*-/- mice (n = 4 per group), with α-tubulin serving as loading control. (D) Isolation of F-actin and G-actin of WT and *Ccnyl1*-/- spermatozoa/testes (n = 2 per group). The F-actin fraction and G-actin fraction were dissolved in an equal volume of buffers, and their contents were examined by western blot. (E) RhoA, Rac1 and Cdc42 activities were measured in testicular lysates of WT and *Ccnyl1*-/- mice (n = 4 per group). The activity was normalized to that of WT mice, which was defined as 1.0. Data are presented as mean ± SEM. (F) Western blotting analysis of p-Ser45/Thr41 β-catenine, p-Ser33/Ser37/Thr41 β-catenine, β-catenine, p-Ser9-Gsk3β and Gsk3β protein levels in testes of WT and *Ccnyl1*-/- mice (n = 4 per group), with β-actin serving as loading control. (G-H) Measurements of intracellular Ca^2+^ and pH levels of germ cells. Mouse germ cells were isolated and co-stained with Hoechst 33342, PI, and (G) Fluo3-AM (Ca^2+^ probe, 1 μM) or (H) BCECF-AM (pH probe, 0.05 μM). PI staining was used to exclude the dead cells, while Hoechst 33342 was used to assign the germ cells into different populations according to their DNA content. 300,000 total cells from each group were examined by FACS analysis. RS: round spermatids; ES: elongating and elongated spermatids. (I) TEM images of seminiferous tubules obtained from testes of adult WT and *Ccnyl1*-/- mice.
(TIF)

S6 Fig Colocalization of CCNYL1 with CDK16 in HEK293T cells. HEK293T cells were co-transfected with CCNYL1*-*HA and CDK16-Flag plasmids for 24 hours. Immunolabeling was performed for analyzing the colocalization of CCNYL1-HA (red) and CDK16-Flag (green). Nuclei were labeled with DAPI (blue). Scale bar: 20 μm.
(TIF)

S7 Fig Further study of phosphorylation modifications on CDK16. (A) WT and K194R CDK16-Flag mutant (kinase dead) were either expressed alone or coexpressed with CCNYL1-HA in HEK293T cells. The interactions were analyzed by CoIP experiments followed by western blotting. For the kinase assay, K194R CDK16-Flag mutant was immuno-precipitated, and incubated with MBP as substrate in kinase buffer. Reaction products were separated by SDS-PAGE and followed by radioautography. (B) WT and S119A CDK16-Flag mutants were co-expressed with CCNYL1-HA in HEK293T cells. The interactions were analyzed by CoIP experiments followed by western blot. (C) Testicular lysates from adult WT mice or cell lysates from HEK293T cells (co-expressed with CCNYL1-HA and CDK16-Flag) were incubated with or without calf intestinal phosphatase (CIP) at 37°C for 30 min and analyzed by western blotting.
(TIF)

S1 Table Mating and progeny production information from WT, *Ccnyl1*+/-, *Ccnyl1*-/- and *Ccny-*/- mice. Healthy two-month-old male WT, *Ccnyl1*+/-, *Ccnyl1*-/- and *Ccny*-/- mice were mated with female mice as indicated in the Table. The fertility status is summarized (—, did not check the vaginal plug of animals in these cages).
(DOC)

S2 Table Analysis of a potential phosphorylation sites on CDK16 by mass spectrometry. CDK16-Flag protein was either expressed alone or together with CCNYL1-HA in HEK293T cells for 24 hours. Samples were collected, and phosphorylation sites on CDK16 were identified by mass spectrometry. In label-free quantification for phosphopeptides, relative comparisons were based on the intensity of extracted ion chromotogram (XIC) from each phosphopeptide, and the average ratio was corrected by protein level. The first nineteen phosphorylation sites were determined with high confidence. The last three, which are labeled in blue, were determined with low confidence.
(DOC)

S3 Table Sequences of primers for real-time PCR. (DOC)

S1 Dataset Phosphorylation sites on CDK16 identified by mass spectrometry. (PDF)
