# Correction: CCNYL1, but Not CCNY, Cooperates with CDK16 to Regulate Spermatogenesis in Mouse

**DOI:** 10.1371/journal.pgen.1008021

**Published:** 2019-03-04

**Authors:** Zhenzhen Zi, Zhuzhen Zhang, Qingrun Li, Weiwei An, Liyong Zeng, Dayuan Gao, Ying Yang, Xueliang Zhu, Rong Zeng, Winnie Waichi Shum, Jiarui Wu

In the Materials and Methods section, the sentence “Ccnyl1tm1a (EUCOMM) Wtsi mice were obtained from the European Conditional Mouse Mutagenesis Program.” should be “The Ccnyl1 knockout-first (kof) mice were kindly provided by EMMA (Stain ID, EM:04396. Stain name, C57BL/6NTac-Ccnyl1<tm1a(EUCOMM)Wtsi>/H, ES cell clone: EPD0177_5_F06) and maintained on a C57BL/6 background.”.

The following sentence should be included in the Acknowledgements section: “The authors would like to thank EMMA for providing the Ccnyl1 knockout-first mice.”.

There is an error in S5 Fig. In S5D Fig, the fourth column along should be Ccnyl1+ and the fifth column along should be Ccnyl1-. Please see the corrected S5 Fig here.

## Supporting information

S5 Fig Deeper characterization of the abnormality of *Ccnyl1-/-* mice. (A) DIC images of spermatozoa collected from caput and cauda epididymidis of adult WT and *Ccnyl1*-/- mice. Black arrow: cytoplasmic droplets, Scale bar: 25 μm. (B) Measurement of β-actin, Cyc (Cytochrome C) and Cox IV (Cytochrome c Oxidase Subunit IV) protein levels of WT and *Ccnyl1*-/- spermatozoa (n = 3 per group), with α-tubulin serving as loading control. (C) Measurement of Cofilin, p-ser3-Cofilin1, Profilin1, Profilin-2 and β-actin protein levels in testis of WT and *Ccnyl1*-/- mice (n = 4 per group), with α-tubulin serving as loading control. (D) Isolation of F-actin and G-actin of WT and *Ccnyl1*-/- spermatozoa/testes (n = 2 per group). The F-actin fraction and G-actin fraction were dissolved in an equal volume of buffers, and their contents were examined by western blot. (E) RhoA, Rac1 and Cdc42 activities were measured in testicular lysates of WT and *Ccnyl1*-/- mice (n = 4 per group). The activity was normalized to that of WT mice, which was defined as 1.0. Data are presented as mean ± SEM. (F) Western blotting analysis of p-Ser45/Thr41 β-catenine, p-Ser33/Ser37/Thr41 β-catenine, β-catenine, p-Ser9-Gsk3β and Gsk3β protein levels in testes of WT and *Ccnyl1*-/- mice (n = 4 per group), with β-actin serving as loading control. (G-H) Measurements of intracellular Ca^2+^ and pH levels of germ cells. Mouse germ cells were isolated and co-stained with Hoechst 33342, PI, and (G) Fluo3-AM (Ca^2+^ probe, 1 μM) or (H) BCECF-AM (pH probe, 0.05 μM). PI staining was used to exclude the dead cells, while Hoechst 33342 was used to assign the germ cells into different populations according to their DNA content. 300,000 total cells from each group were examined by FACS analysis. RS: round spermatids; ES: elongating and elongated spermatids. (I) TEM images of seminiferous tubules obtained from testes of adult WT and *Ccnyl1*-/- mice.
(TIF)

## References

[1] Zi Z, Zhang Z, Li Q, An W, Zeng L, Gao D, et al (2015) CCNYL1, but Not CCNY, Cooperates with CDK16 to Regulate Spermatogenesis in Mouse. PLOS Genetics 11(8): e1005485 10.1371/journal.pgen.1005485 26305884 PMC4549061

