# Supplementary figures and images for: CCNYL1, but Not CCNY, Cooperates with CDK16 to Regulate Spermatogenesis in Mouse
Source: PLoS Genet. 2015 Aug 25;11(8):e1005485. doi: 10.1371/journal.pgen.1005485 (PMC4549061; doi:10.1371/journal.pgen.1005485)

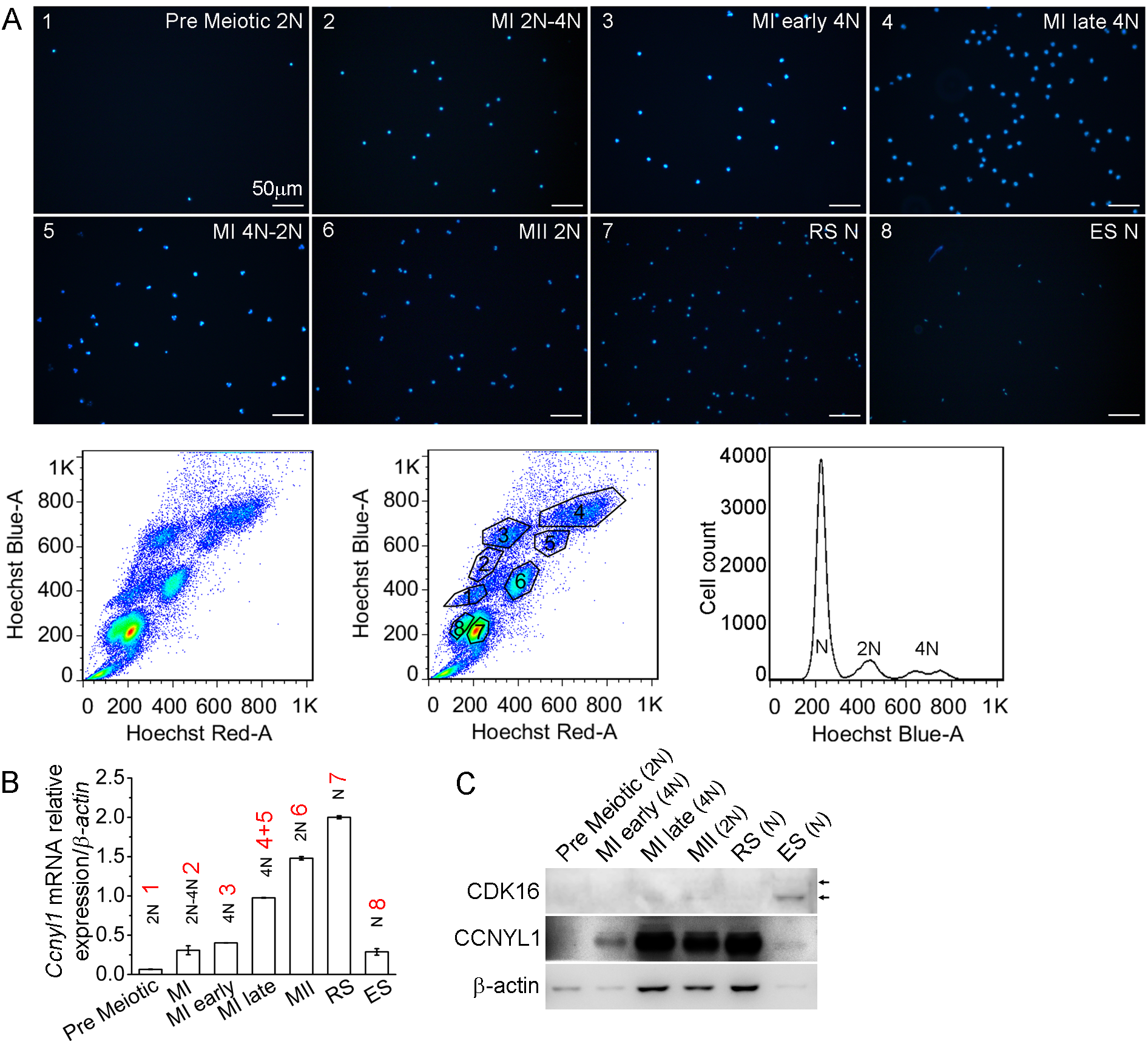

Supplement: S1 Fig — (A) Mouse germinal cells were isolated by the FACS sorting method. Propidium iodide (PI) staining was used to exclude the dead cells, while Hoechst 33342 was used to assign the cells into different populations according to their DNA content. (MI: Meiosis I; MII: Meiosis II; RS: Round Spermatids; ES: Elongating and Elongated Spermatids; N: Haploid; 2N; Diploid; 4N: Tetraploid). The fluorescence images were taken just after sorting (Olympus IX71 microscopy). (B) Ccnyl1 mRNA levels were measured in sorted germinal cell populations from a WT testis. (MI: Meiosis I; MII: Meiosis II; RS: Round Spermatids; ES: Elongating and Elongated Spermatids; N: Haploid; 2N; Diploid; 4N: Tetraploid). The cell populations are also indicated. Data are presented as mean ± SEM. (C) CCNYL1 and CDK16 protein levels were measured in sorted germinal cells by western blotting. (TIF) [file pgen.1005485.s001.tif]

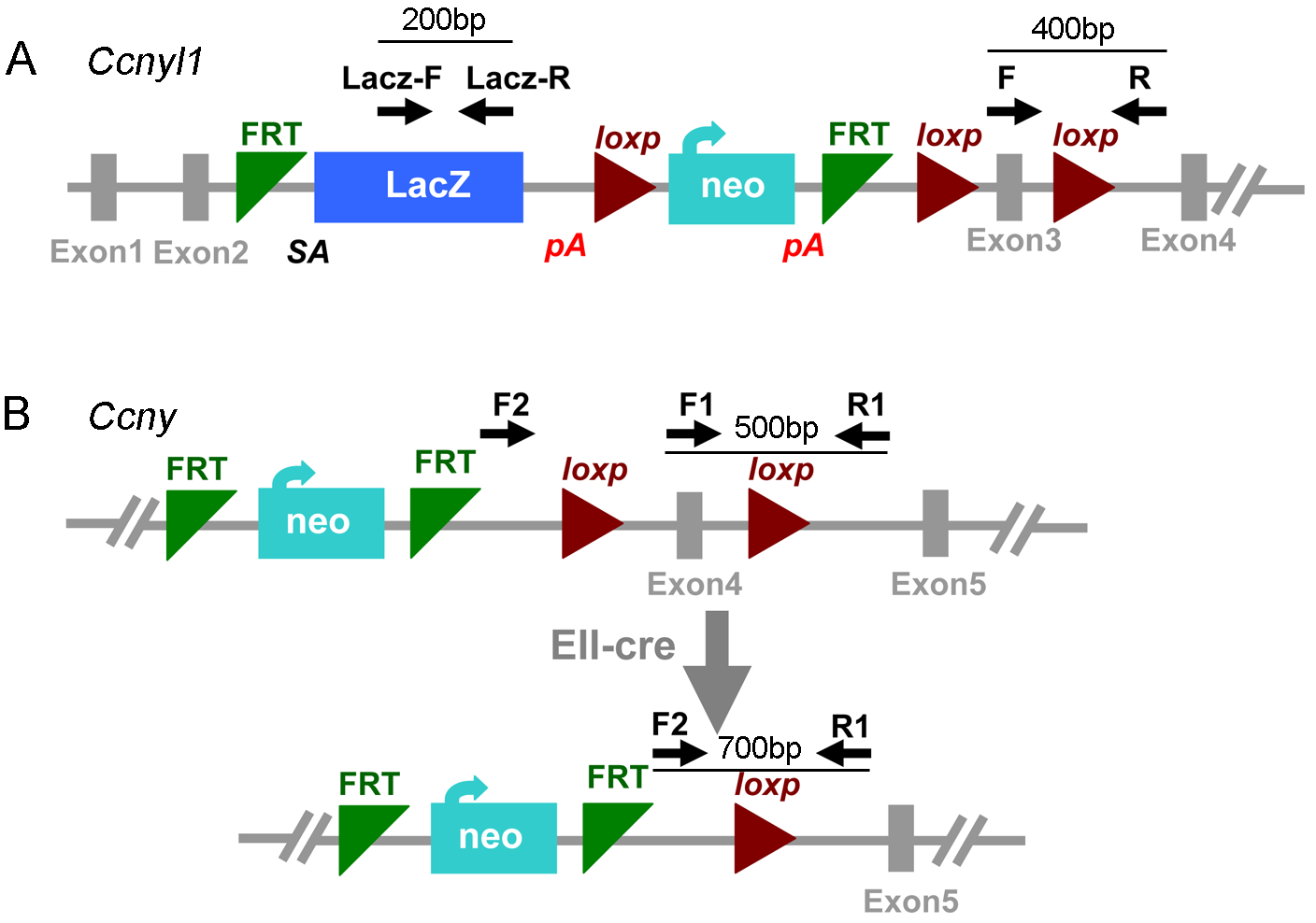

Supplement: S2 Fig — (A) Structure of targeted Ccnyl1 locus. The black arrow depicts the primers for genotyping. Ccnyl1 knockout mice were first knockout with stop codon and polyA tails following LacZ sequences. The gray boxes depict the Ccnyl1 exons. The neomycin resistance cassette is shown as a light blue box, the loxP sites are shown as red triangles, and the FRT sites are shown as green triangles. (B) Structure of targeted Ccny locus. The black arrow depicts the primers for genotyping, and the exons of Ccny are shown as gray boxes. (TIF) [file pgen.1005485.s002.tif]

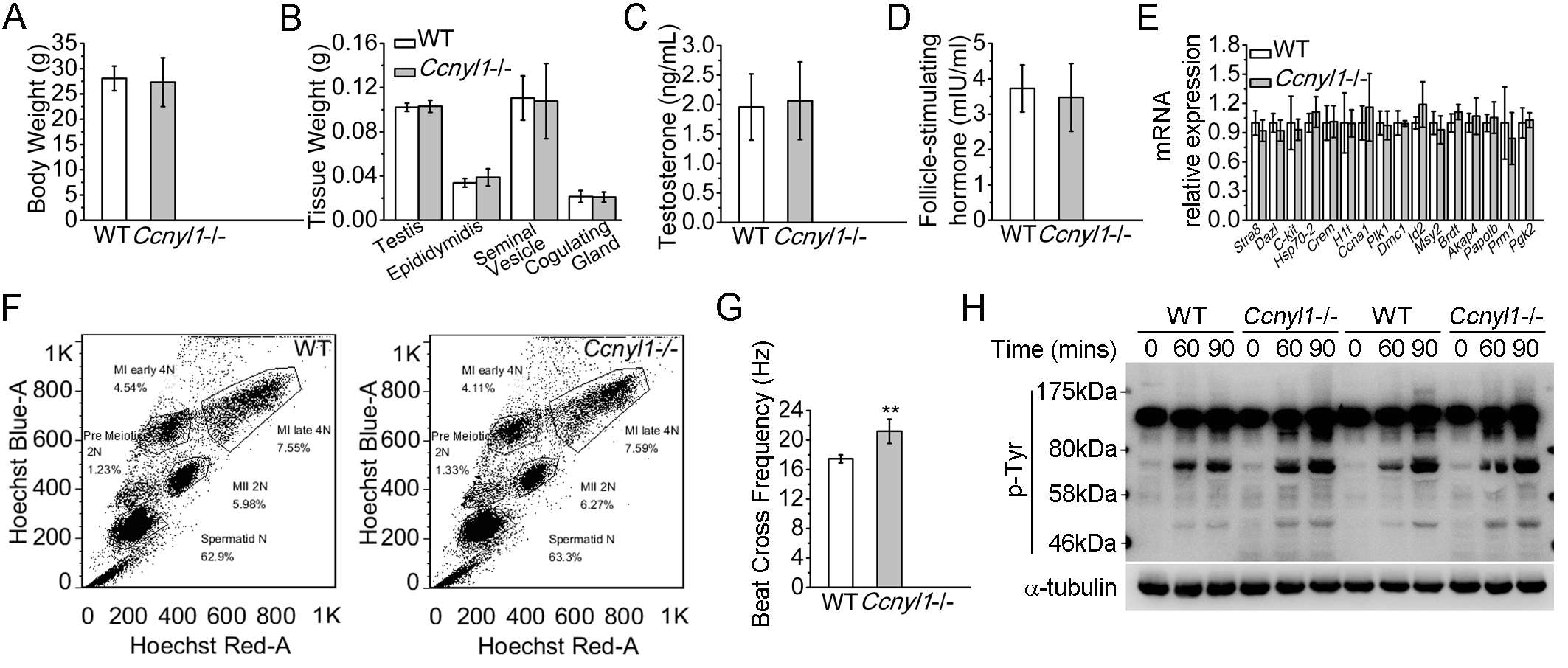

Supplement: S3 Fig — (A-D) Body weight (A), reproductive organ weight (B), serum testosterone (C) and follicle-stimulating hormone (D) of male WT and Ccnyl1-/- mice (n ≥ 5 per group). Data are presented as mean ± SEM for bar graphs and hereafter. (E) Measurement of genes specifically expressed in different germ cell populations of WT and Ccnyl1-/- mice (n = 5 per group). Gene expression level was normalized to WT mice, which was defined as 1.0. (F) FACS analysis of the germ cell populations between adult WT and Ccnyl1-/- mice. (G) Beat cross frequency of spermatozoa collected from the cauda epididymidis of WT and Ccnyl1-/- mice (n = 5 per group). *P < 0.05; **P < 0.01. (H) Spermatozoa collected from the cauda epididymidis of WT and Ccnyl1-/- mice were incubated in full EKRB buffer (with Ca2+, BSA, NaHCO3) for capacitation. Spermatozoa were collected at 0, 60, and 180 minutes after incubation (n = 2 per group). Tyrosine phosphorylation levels were measured by western blotting, with α-tubulin serving as loading control. (TIF) [file pgen.1005485.s003.tif]

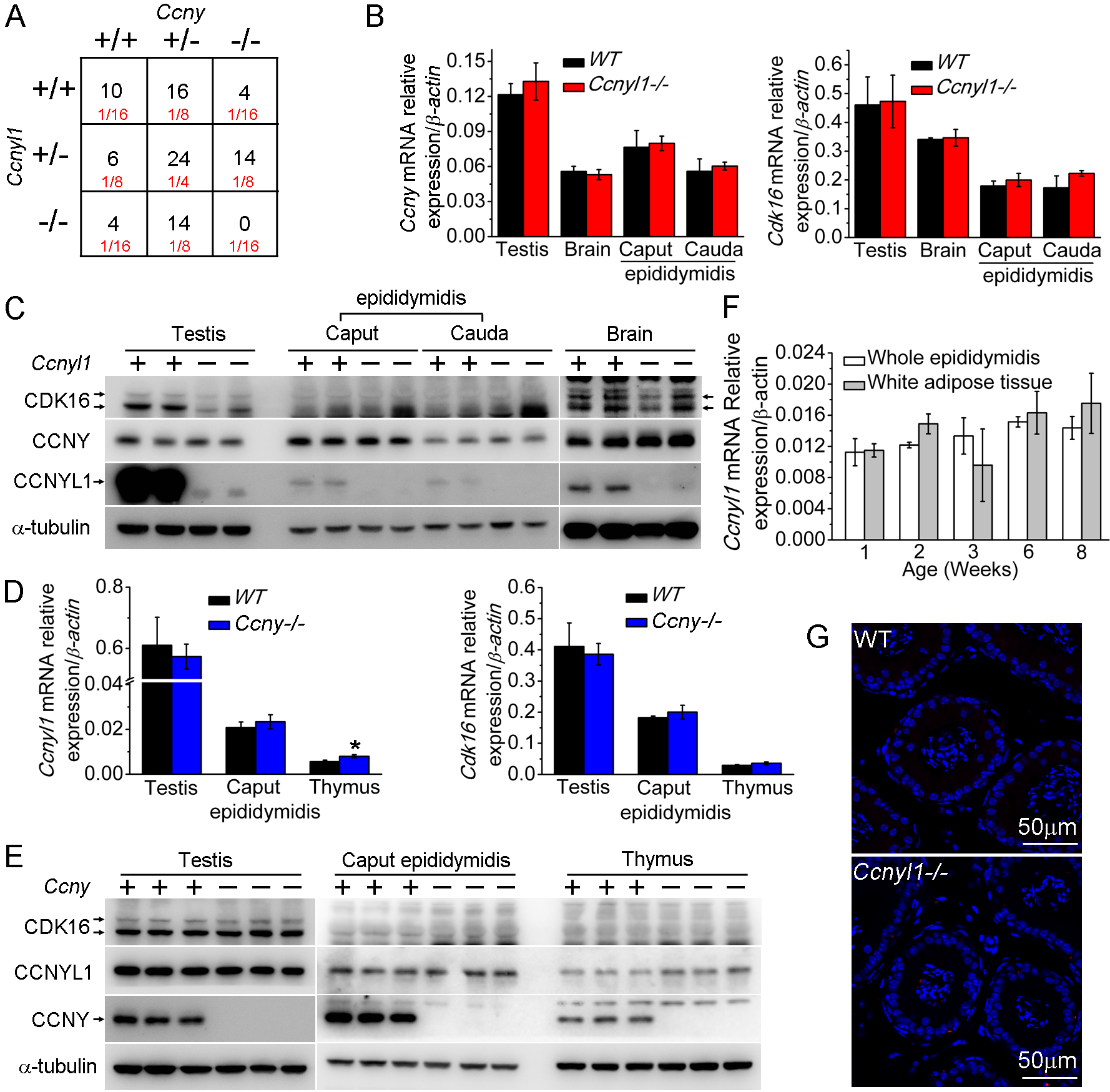

Supplement: S4 Fig — (A) Male and female Ccnyl1+/-Ccny+/- mice were intercrossed, numbers of offspring with different genotypes were counted (total: 92 mice). The red numbers are the theoretical distribution according to Mendel's laws of inheritance. (B-C) Both mRNA and protein levels of CCNY expressions were measured in the testis, brain, and caput/cauda epididymidis of adult Ccnyl1-/- and WT mice (mRNA: n = 5/group, protein: n = 2-3/group). (D-E) Both mRNA and protein levels of CCNYL1 expressions were measured in the testis, brain, and caput/cauda epididymidis of adult Ccny-/- and WT mice (mRNA: n = 5/group, protein: n = 2-3/group). (F) mRNA levels of Ccnyl1 expression were measured in whole epididymides and white adipose tissues of mice at different ages (n = 4). (G) Immunolabeling of CCNYL1 (red) in sections of the epididymis. Sections from Ccnyl1-/- epididymis were stained as the negative control. Nuclei were labeled with DAPI (blue). (TIF) [file pgen.1005485.s004.tif]

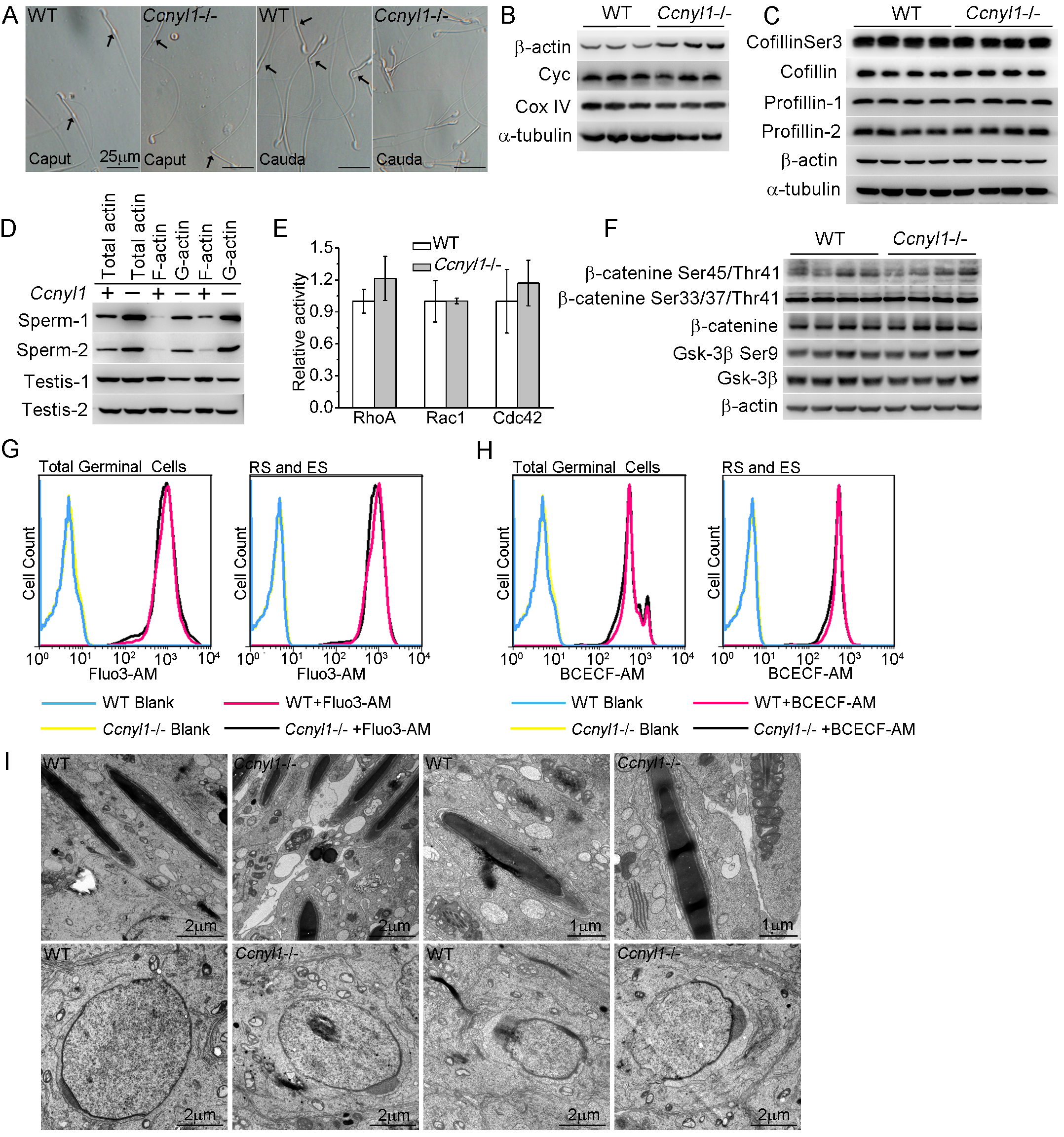

Supplement: S5 Fig — (A) DIC images of spermatozoa collected from caput and cauda epididymidis of adult WT and Ccnyl1-/- mice. Black arrow: cytoplasmic droplets, Scale bar: 25 μm. (B) Measurement of β-actin, Cyc (Cytochrome C) and Cox IV (Cytochrome c Oxidase Subunit IV) protein levels of WT and Ccnyl1-/- spermatozoa (n = 3 per group), with α-tubulin serving as loading control. (C) Measurement of Cofilin, p-ser3-Cofilin1, Profilin1, Profilin-2 and β-actin protein levels in testis of WT and Ccnyl1-/- mice (n = 4 per group), with α-tubulin serving as loading control. (D) Isolation of F-actin and G-actin of WT and Ccnyl1-/- spermatozoa/testes (n = 2 per group). The F-actin fraction and G-actin fraction were dissolved in an equal volume of buffers, and their contents were examined by western blot. (E) RhoA, Rac1 and Cdc42 activities were measured in testicular lysates of WT and Ccnyl1-/- mice (n = 4 per group). The activity was normalized to that of WT mice, which was defined as 1.0. Data are presented as mean ± SEM. (F) Western blotting analysis of p-Ser45/Thr41 β-catenine, p-Ser33/Ser37/Thr41 β-catenine, β-catenine, p-Ser9-Gsk3β and Gsk3β protein levels in testes of WT and Ccnyl1-/- mice (n = 4 per group), with β-actin serving as loading control. (G-H) Measurements of intracellular Ca2+ and pH levels of germ cells. Mouse germ cells were isolated and co-stained with Hoechst 33342, PI, and (G) Fluo3-AM (Ca2+ probe, 1 μM) or (H) BCECF-AM (pH probe, 0.05 μM). PI staining was used to exclude the dead cells, while Hoechst 33342 was used to assign the germ cells into different populations according to their DNA content. 300,000 total cells from each group were examined by FACS analysis. RS: round spermatids; ES: elongating and elongated spermatids. (I) TEM images of seminiferous tubules obtained from testes of adult WT and Ccnyl1-/- mice. (TIF) [file pgen.1005485.s005.tif]

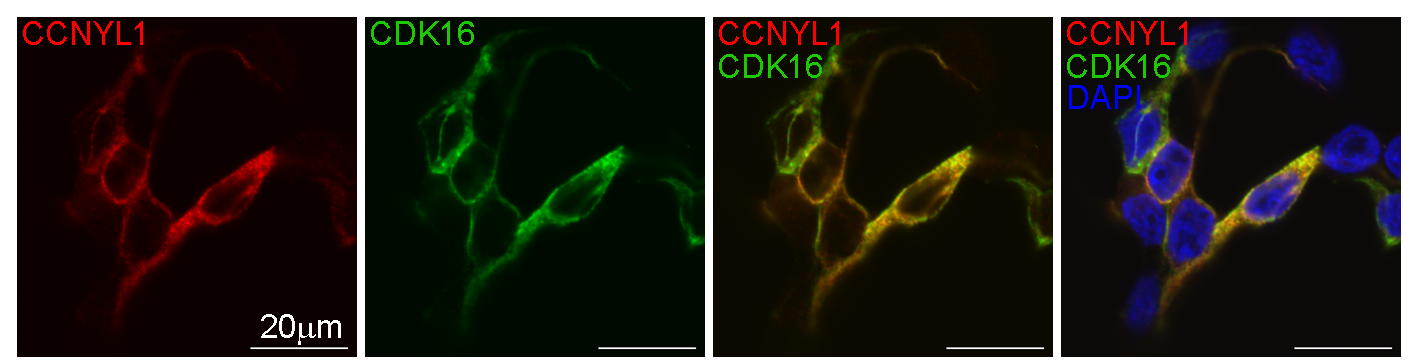

Supplement: S6 Fig — HEK293T cells were co-transfected with CCNYL1-HA and CDK16-Flag plasmids for 24 hours. Immunolabeling was performed for analyzing the colocalization of CCNYL1-HA (red) and CDK16-Flag (green). Nuclei were labeled with DAPI (blue). Scale bar: 20 μm. (TIF) [file pgen.1005485.s006.tif]

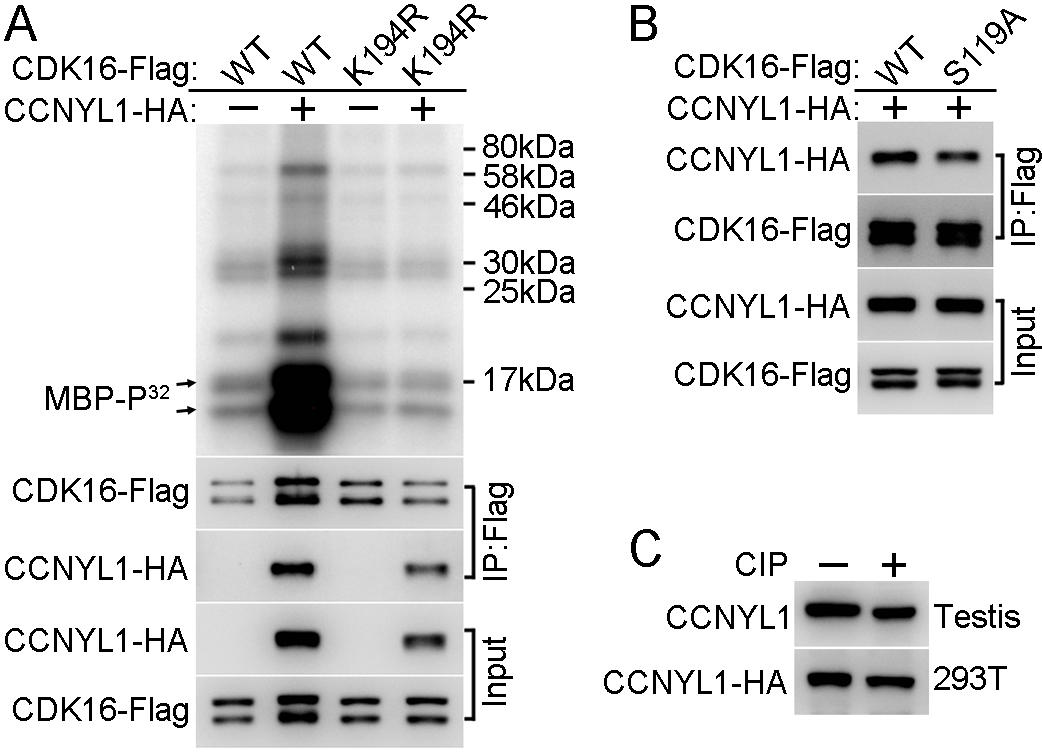

Supplement: S7 Fig — (A) WT and K194R CDK16-Flag mutant (kinase dead) were either expressed alone or coexpressed with CCNYL1-HA in HEK293T cells. The interactions were analyzed by CoIP experiments followed by western blotting. For the kinase assay, K194R CDK16-Flag mutant was immuno-precipitated, and incubated with MBP as substrate in kinase buffer. Reaction products were separated by SDS-PAGE and followed by radioautography. (B) WT and S119A CDK16-Flag mutants were co-expressed with CCNYL1-HA in HEK293T cells. The interactions were analyzed by CoIP experiments followed by western blot. (C) Testicular lysates from adult WT mice or cell lysates from HEK293T cells (co-expressed with CCNYL1-HA and CDK16-Flag) were incubated with or without calf intestinal phosphatase (CIP) at 37°C for 30 min and analyzed by western blotting. (TIF) [file pgen.1005485.s007.tif]
