# Supplementary material for: CCNYL1, but Not CCNY, Cooperates with CDK16 to Regulate Spermatogenesis in Mouse
Source: PLoS Genet. 2015 Aug 25;11(8):e1005485. doi: 10.1371/journal.pgen.1005485 (PMC4549061; doi:10.1371/journal.pgen.1005485)
Supplement: S1 Table — Healthy two-month-old male WT, Ccnyl1+/-, Ccnyl1-/- and Ccny-/- mice were mated with female mice as indicated in the Table. The fertility status is summarized (—, did not check the vaginal plug of animals in these cages). (DOC) [file pgen.1005485.s008.doc]

**Table S1. Mating and progeny production information from WT, *Ccnyl1*+/-, *Ccnyl1*-/- and *Ccny-*/- mice.**

| Genotype of female | Genetype of male | Mating ratio (Female/Male) | Vaginal plug | Mating days | Pregnant female |
| --- | --- | --- | --- | --- | --- |
| WT | *Ccnyl1*-/- | 3:1 | Detected | 65 | 0 |
| WT | *Ccnyl1*-/- | 3:1 | Detected | 65 | 0 |
| WT | *Ccnyl1*-/- | 3:1 | Detected | 25 | 0 |
| WT | *Ccnyl1*-/- | 1:1 | -- | 46 | 0 |
| WT | *Ccnyl1*-/- | 3:1 | -- | 32 | 0 |
| WT | *Ccnyl1*-/- | 3:1 | -- | 57 | 0 |
| *Ccnyl1*+/- | *Ccnyl1*+/- | 1:1 | -- | 22 | Born 8 mice |
| *Ccnyl1*+/- | *Ccnyl1*+/- | 1:1 | -- | 21 | Born 9 mice |
| *Ccnyl1*+/- | *Ccnyl1*+/- | 1:1 | -- | 22 | Born 8 mice |
| *Ccnyl1*+/- | *Ccnyl1*+/- | 1:1 | -- | 22 | Born 6 mice |
| *Ccnyl1*+/- | *Ccnyl1*+/- | 1:1 | -- | 22 | Born 10 mice |
| *Ccnyl1*+/- | *Ccnyl1*+/- | 1:1 | -- | 21 | Born 8 mice |
| *Ccnyl1*-/- | WT | 3:1 | -- | 15 | 2 |
| *Ccnyl1*-/- | WT | 3:1 | -- | 15 | 3 |
| *Ccnyl1*-/- | WT | 1:1 | -- | 21 | Born 11 mice |
| *Ccnyl1*-/- | WT | 1:1 | -- | 21 | Born 9 mice |
| *Ccny*-/- | *Ccny*-/- | 1:1 | -- | 21 | Born 8 mice |
| *Ccny*-/- | *Ccny*-/- | 1:1 | -- | 23 | Born 5 mice |
| *Ccny*-/- | *Ccny*-/- | 1:1 | -- | 22 | Born 9 mice |
| *Ccny*-/- | *Ccny*-/- | 1:1 | -- | 22 | Born 7 mice |
