# Supplementary material for: CCNYL1, but Not CCNY, Cooperates with CDK16 to Regulate Spermatogenesis in Mouse
Source: PLoS Genet. 2015 Aug 25;11(8):e1005485. doi: 10.1371/journal.pgen.1005485 (PMC4549061; doi:10.1371/journal.pgen.1005485)
Supplement: S2 Table — CDK16-Flag protein was either expressed alone or together with CCNYL1-HA in HEK293T cells for 24 hours. Samples were collected, and phosphorylation sites on CDK16 were identified by mass spectrometry. In label-free quantification for phosphopeptides, relative comparisons were based on the intensity of extracted ion chromotogram (XIC) from each phosphopeptide, and the average ratio was corrected by protein level. The first nineteen phosphorylation sites were determined with high confidence. The last three, which are labeled in blue, were determined with low confidence. (DOC) [file pgen.1005485.s009.doc]

**Table S2. Analysis of a potential phosphorylation sites on CDK16 by mass spectrometry.**

| Protein names | Phospho (STY) Probabilities | Position in protein | Intensity (CDK16+CCNYL1) | Intensity  (CDK16) | Average Ratio (CDK16+CCNYL1)  /(CDK16) |
| --- | --- | --- | --- | --- | --- |
| CDK16 | QLS(1)MTLR | 12 | 10433220000 | 4102780000 | 1.33 |
| CDK16 | TNGVPEQIGLDES(0.999)GGGGGS(0.001)DLGEAPTR | 36 | 37636800 | 0 | ↑ |
| CDK16 | GPLS(0.973)S(0.027)APEIVHEDMK | 64 | 0 | 0 | 0 |
| CDK16 | GPLSS(1)APEIVHEDMK | 65 | 511190000 | 390405000 | 0.69 |
| CDK16 | MGS(1)DGESDQASAT(0.021)S(0.47)S(0.387)DEVQS(0.121)PVRVR | 78 | 516772000 | 3161660000 | 0.09↓ |
| CDK16 | GPLSS(0.001)APEIVHEDMKMGS(0.184)DGES(0.808)DQAS(0.005)ATSS  DEVQSPVR | 82 | 0 | 0 | 0 |
| CDK16 | MGSDGESDQASAT(0.04)S(0.784)S(0.176)DEVQSPVR | 89 | 36905500 | 27612750 | 0.8 |
| CDK16 | MGSDGESDQASATSSDEVQS(1)PVR | 95 | 351650000 | 101680000 | 1.8 |
| CDK16 | KIS(1)TEDINKR | 110 | 1666320000 | 870882000 | 1 |
| CDK16 | RLS(1)LPADIRLPEGYLEK | 119 | 2851320000 | 1805760000 | 0.82 |
| CDK16 | LTLNS(1)PIFDKPLSR | 138 | 7046254000 | 4118894000 | 0.85 |
| CDK16 | LT(0.001)LNS(0.999)PIFDKPLS(1)RR | 146 | 578412000 | 16570000 | 18.4↑ |
| CDK16 | RVS(1)LSEIGFGK | 153 | 15401700000 | 10440060000 | 0.78 |
| CDK16 | VSLS(1)EIGFGK | 155 | 140706000 | 168481000 | 0.37 |
| CDK16 | LGEGT(1)YATVYK | 175 | 19070400 | 0 | ↑ |
| CDK16 | ILGT(0.953)PT(0.047)EETWPGILSNEEFR | 380 | 7255600 | 1991340 | 1.96 |
| CDK16 | ILGTPTEETWPGILS(1)NEEFR | 391 | 198900000 | 32512000 | 3.2 |
| CDK16 | EANIRS(0.792)T(0.196)S(0.012)MPDSGRPAFR | 478 | 0 | 0 | 0 |
| CDK16 | STS(1)MPDSGRPAFR | 480 | 3355140000 | 274950000 | 6.4↑ |
| CDK16 | GPLSSAPEIVHEDMKMGS(0.004)DGES(0.074)DQAS(0.208)AT(0.208)  S(0.208)S(0.174)DEVQS(0.123)PVRVR | 86 | 0 | 0 | 0 |
| CDK16 | MGS(1)DGESDQASAT(0.012)S(0.139)S(0.449)DEVQS(0.399)PVRVR | 90 | 0 | 0 | 0 |
| CDK16 | MGS(0.998)DGES(0.004)DQAS(0.131)AT(0.249)S(0.225)S(0.202)DEV  QS(0.19)PVRVR | 88 | 0 | 0 | 0 |
