# Supplementary material for: CCNYL1, but Not CCNY, Cooperates with CDK16 to Regulate Spermatogenesis in Mouse
Source: PLoS Genet. 2015 Aug 25;11(8):e1005485. doi: 10.1371/journal.pgen.1005485 (PMC4549061; doi:10.1371/journal.pgen.1005485)
Supplement: S3 Table — (DOC) [file pgen.1005485.s010.doc]

**Table S3.** Sequences of primers for Real-time PCR

| Gene name | Forward primer | Reverse primer |
| --- | --- | --- |
| *Ccnyl1* | GCGGTCACATCCTCTCACAA | ACATTCCGCTGTCAGCTGTG |
| *Ccny* | ACAGCTCTTGCTCCACCATC | GCATCCTTCCATCTGGGTCC |
| *CDK5* | AAGCCCTACCCAATGTACCC | GTCAGAGAAGTAGGGGTGCT |
| *CDK14* | TTGTCCGAGAGTTTCAGCCG | TCAAAAGTGCTCTGCGTCCT |
| *CDK15* | AGGGGTTGCCATGGAAGAAG | AGGAGAGATGCTTCCCGGAT |
| *CDK16* | GTCGCAATCGGATCTCTGCT | TCGGTATCCACCACACGGAA |
| *CDK17* | GTTGCACCGGGACTTGAAAC | GAACTGACTTGGCTCTGGCT |
| *CDK18* | CGGGCTCTACTGTCAAGGAG | CTGTGGTAGGTATCGGGGGA |
| *Gapdh* | TGGCAAAGTGGAGATTGTTGCC | AAGATGGTGATGGGCTTCCCG |
| β-actin | AAATCGTGCGTGACATCAAAGA | GCCATCTCCTGCTCGAAGTC |
| *Dazl* | GAAGTCTGTGGACCGAAGCA | CACTGCCCGACTTCTTCTGA |
| *C-kit* | TGACGGTACATGGCTGCATT | ACCACCGTAAATGTGTCCCC |
| *Hsp70-2* | TAACCAACGACAAGGGTCGG | GTCTTCCACGGTCTGCTTGA |
| *Crem* | CTTTGCCACAAGGTGTGGTG | CTTCTTCCTGCGACACTCCC |
| *Akap4* | GTCAGAAGGCGAGTTAAATCTGG | ATCCCTCCGTCTTAGACTGGT |
| *Brdt* | GCTTTGGGACTCCACAACTACTATG | GATTGTCCATTTTCCCCTTGATC |
| *Ccna1* | TTTCCCCAATGCTGGTTGA | AACCAAAATCCGTTGCTTCCT |
| *H1t* | GCTGATTCCTGAGGCCCTTT | CAGGGCAGCAAGGGACAT |
| *Msy2* | CATCCTTATTGTTCCGAGGCA | GGAGGTATGAGCTGGCTGGTT |
| *Papolb* | CGCCAACAGAGAAACAACATTTAG | CCAACCAGGATTCGGATCTTT |
| *Plk1* | CGAGGATCTGGAGGTGAAAA | AGGAGTGCCACACAAGGTCT |
| *Stra8* | GAGTGAGGCCCAGCATATGTC | CCTCTGGATTTTCTGAGTTGCA |
| *Dmc1* | ATGAAGGAGGATCAAGTTGTGC | CATGCTTCTGCAACAGGTCAA |
| *Id2* | ATGAAAGCCTTCAGTCCGGTG | AGCAGACTCATCGGGTCGT |
| *Prm1* | CCGTCGCAGACGAAGATGTC | CACCTTATGGTGTATGAGCGG |
| *Pgk2* | TTCTGCTAAGTTGACTCTGGACA | AGCCTTGATTCTCTGGTTGTTTG |
