# Supplementary material for: CCNYL1, but Not CCNY, Cooperates with CDK16 to Regulate Spermatogenesis in Mouse
Source: PLoS Genet. 2015 Aug 25;11(8):e1005485. doi: 10.1371/journal.pgen.1005485 (PMC4549061; doi:10.1371/journal.pgen.1005485)

Raw file	Scan	Method	Score	m/z	Gene names
F_20141230_JRWu_ZZZheng_Pho_2_UM1	16926	FTMS; HCD	148.5	464.72	Cdk16

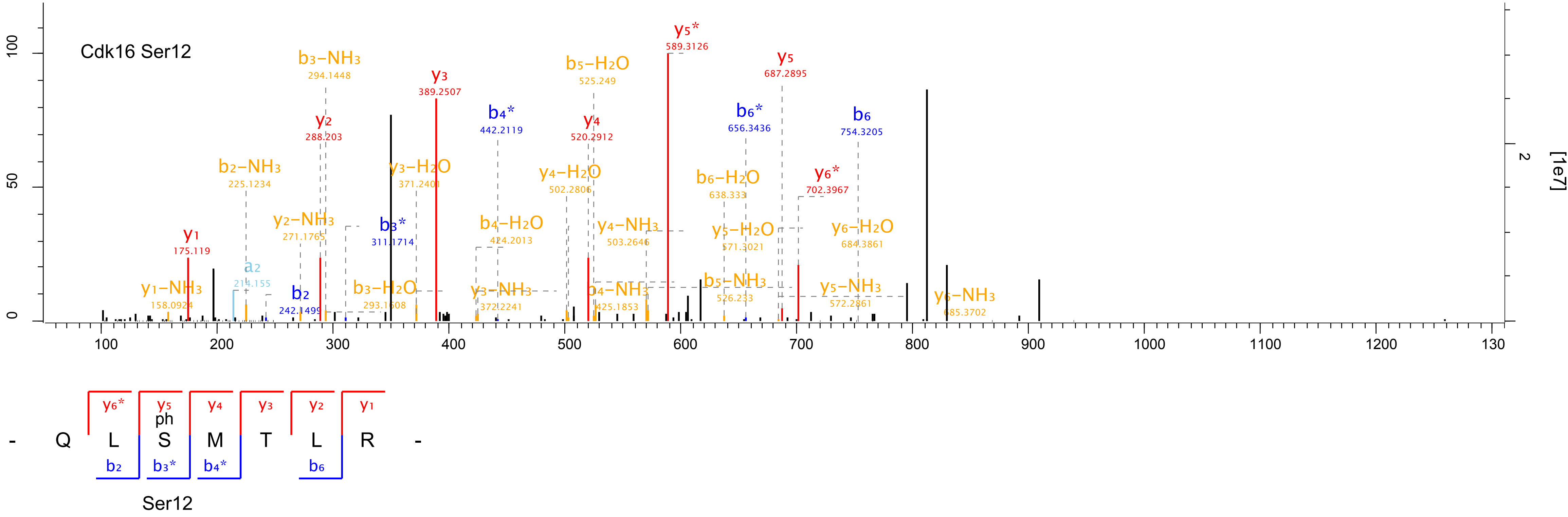

Raw file	Scan	Method	Score	m/z	Gene names
F_20141230_JRWu_ZZZheng_Pho_2_double-1	16218	FTMS; HCD	84.48	884.39	Cdk16

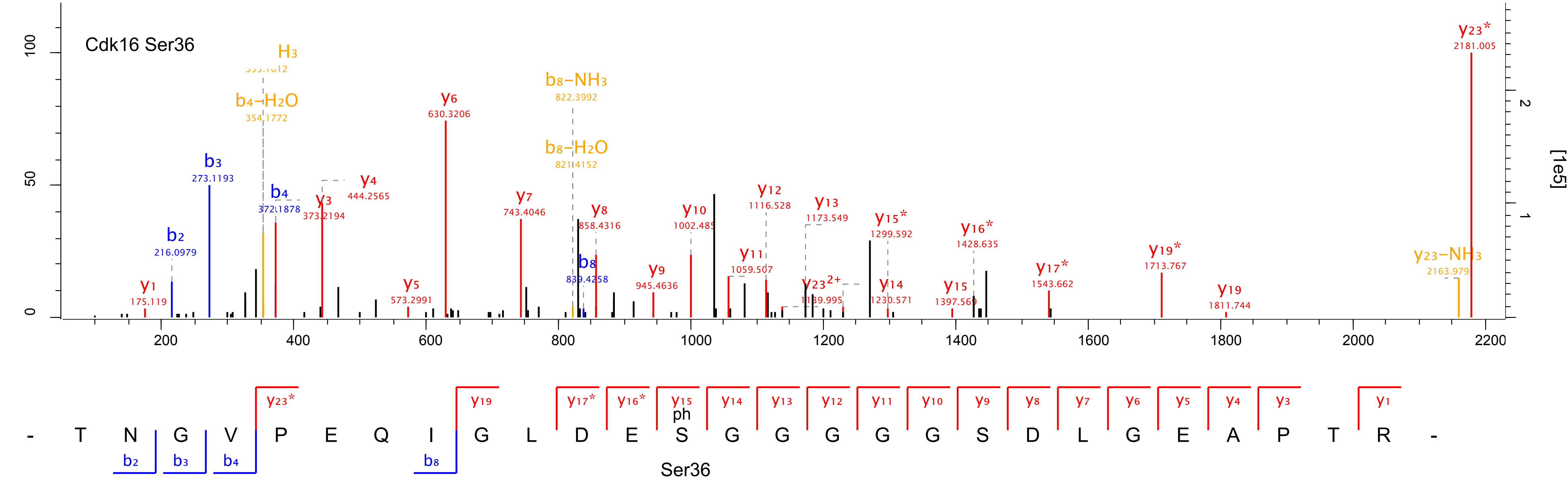

Raw file	Scan	Method	Score	m/z	Gene names
F_20141230_JRWu_ZZZheng_Pho_2_double-3	17089	FTMS; HCD	149.47	845.38	Cdk16

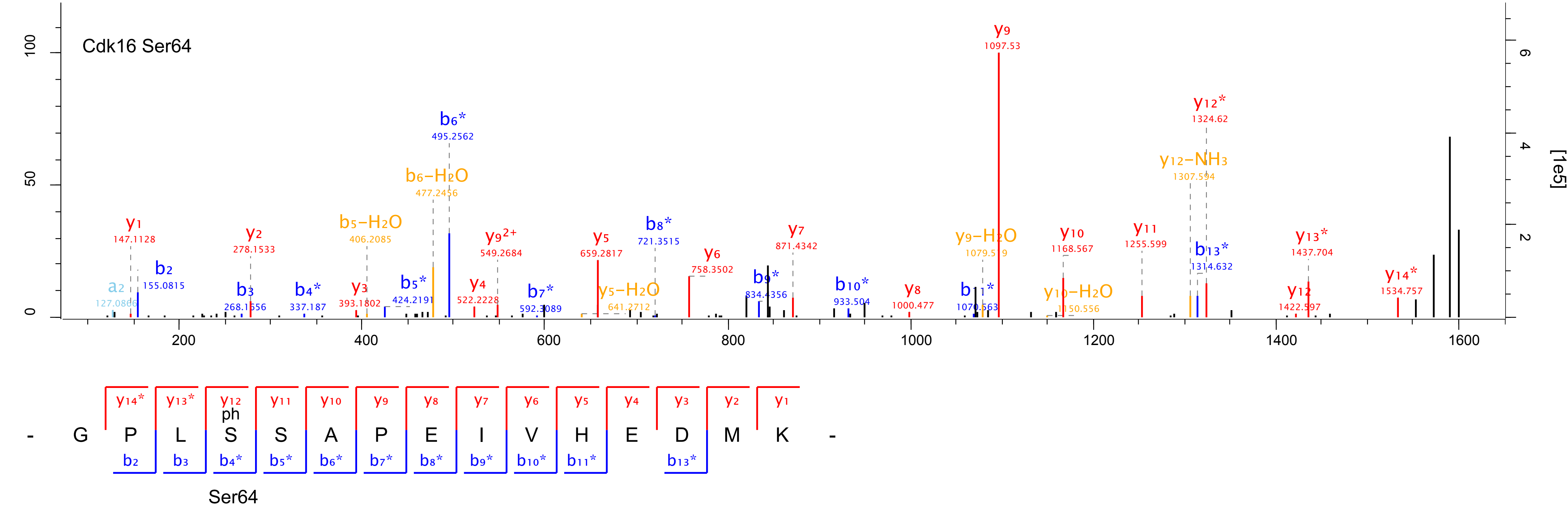

Raw file	Scan	Method	Score	m/z	Gene names
F_20141230_JRWu_ZZZheng_Pho_2_double-3	15048	FTMS; HCD	223.45	845.38	Cdk16

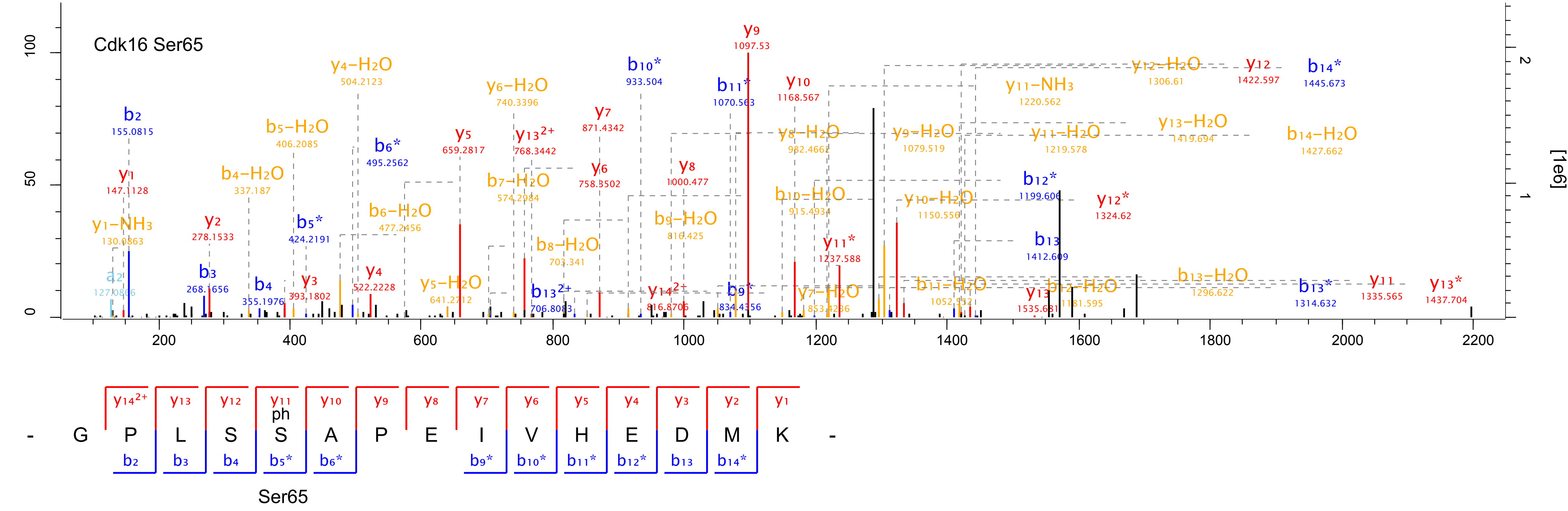

Raw file	Scan	Method	Score	m/z	Gene names
F_20141230_JRWu_ZZZheng_Pho_1_single-2	10754	FTMS; HCD	155.55	919.03	Cdk16

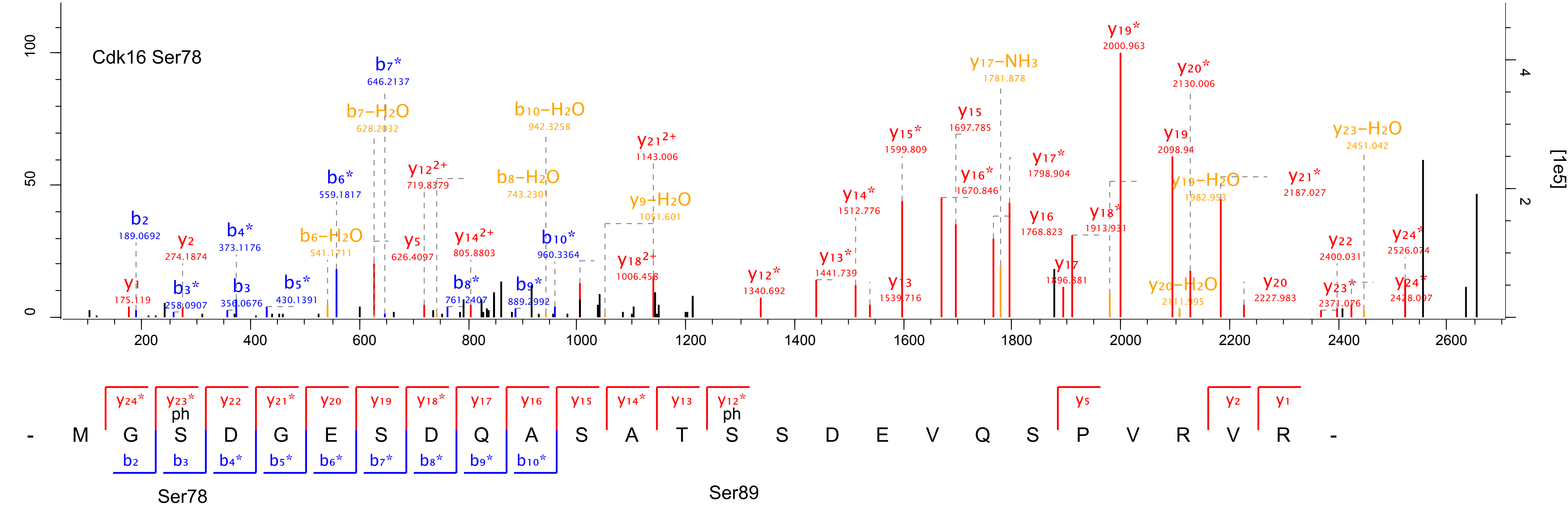

Raw file	Scan	Method	Score	m/z	Gene names
F_20141230_JRWu_ZZZheng_Pho_1_single-2	16263	FTMS; HCD	83.36	1007.94	Cdk16

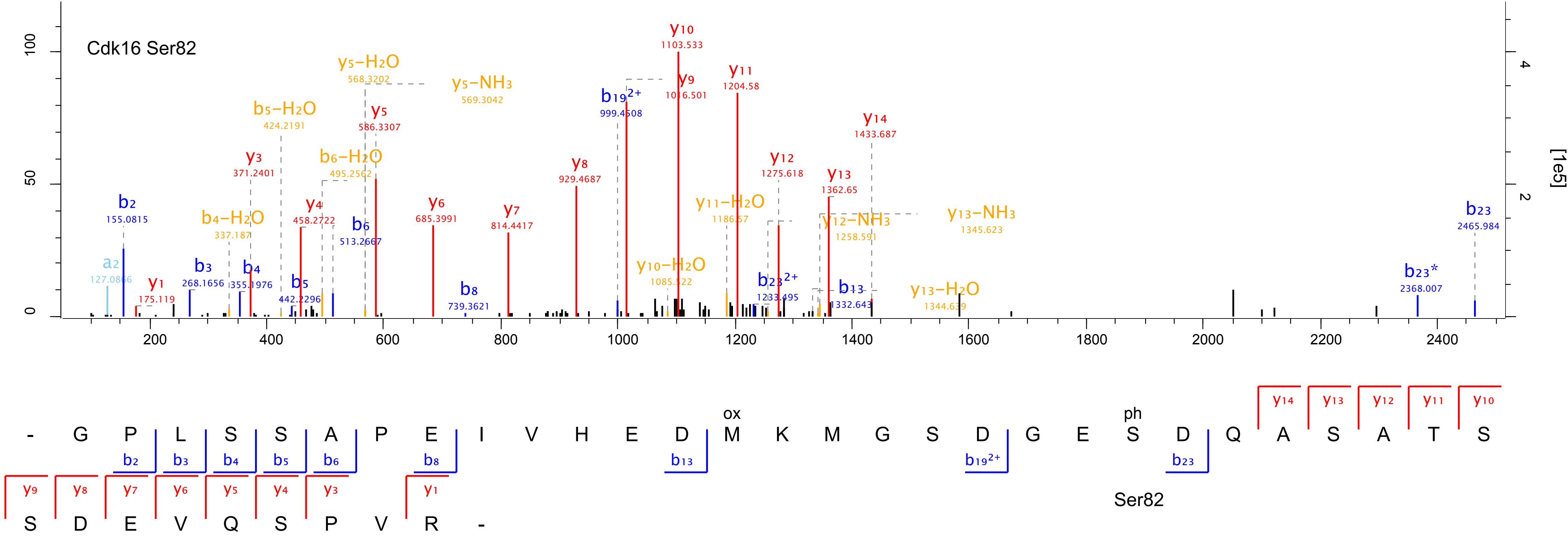

Raw file	Scan	Method	Score	m/z	Gene names
F_20141219_JRWu_ZZZheng_Pho_2_double-1	9577	FTMS; HCD	142.65	1210.98	Cdk16

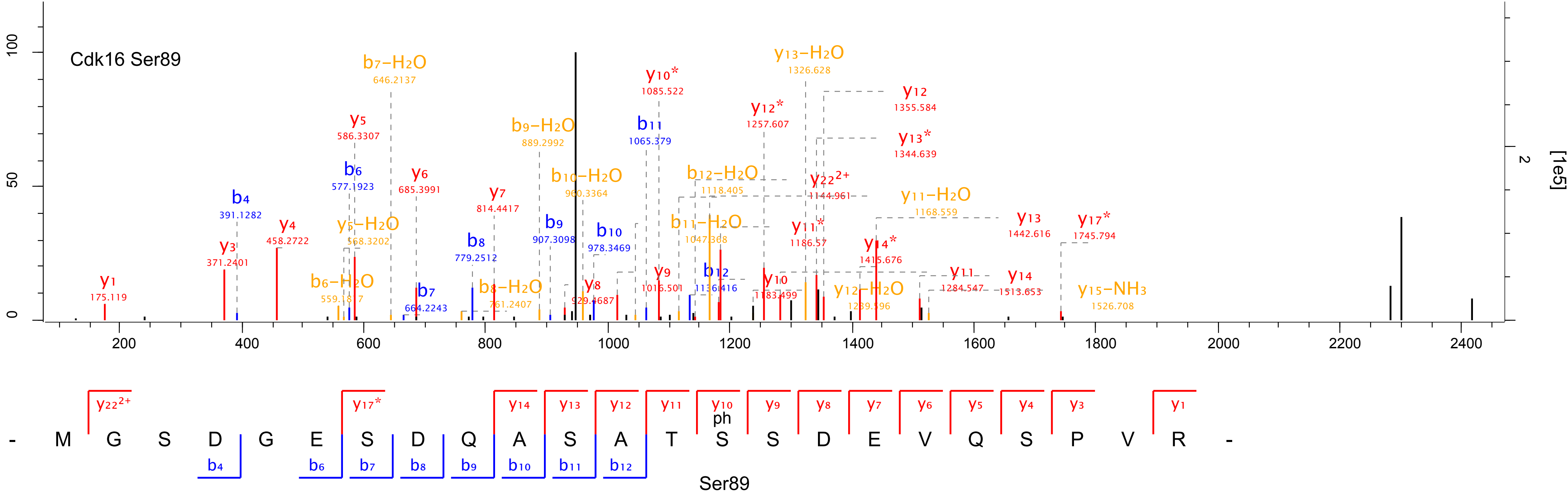

Raw file	Scan	Method	Score	m/z	Gene names
F_20141219_JRWu_ZZZheng_Pho_2_double-3	7590	FTMS; HCD	216.38	807.66	Cdk16

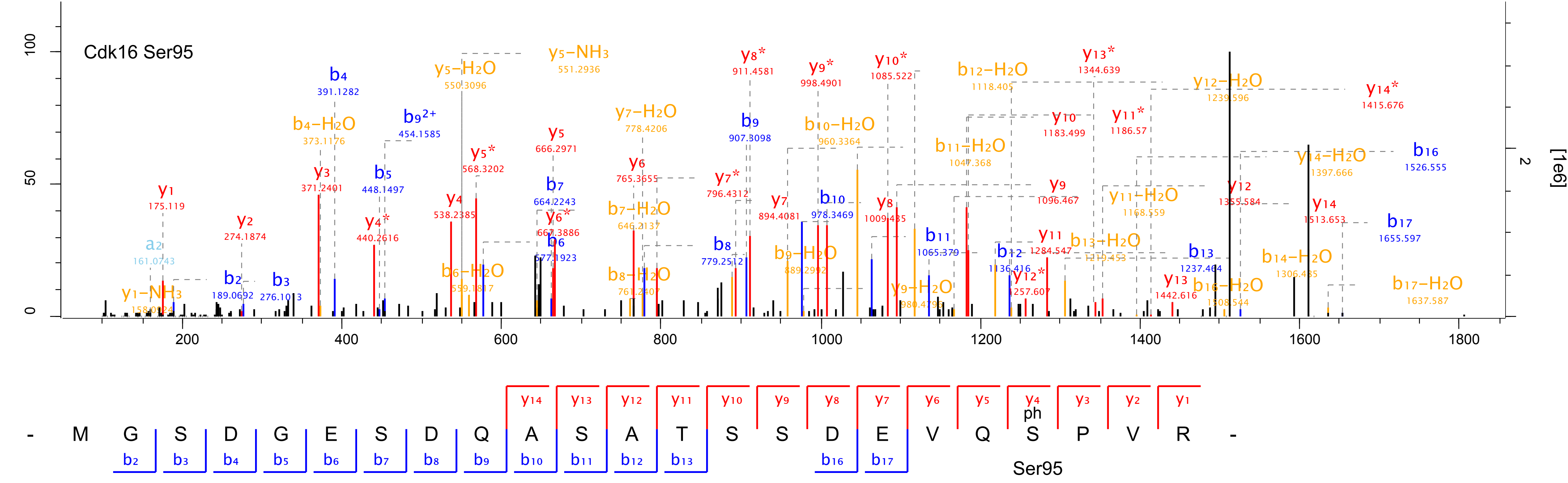

Raw file	Scan	Method	Score	m/z	Gene names
F_20141219_JRWu_ZZZheng_Pho_2_double-1	4141	FTMS; HCD	242.91	642.32	Cdk16

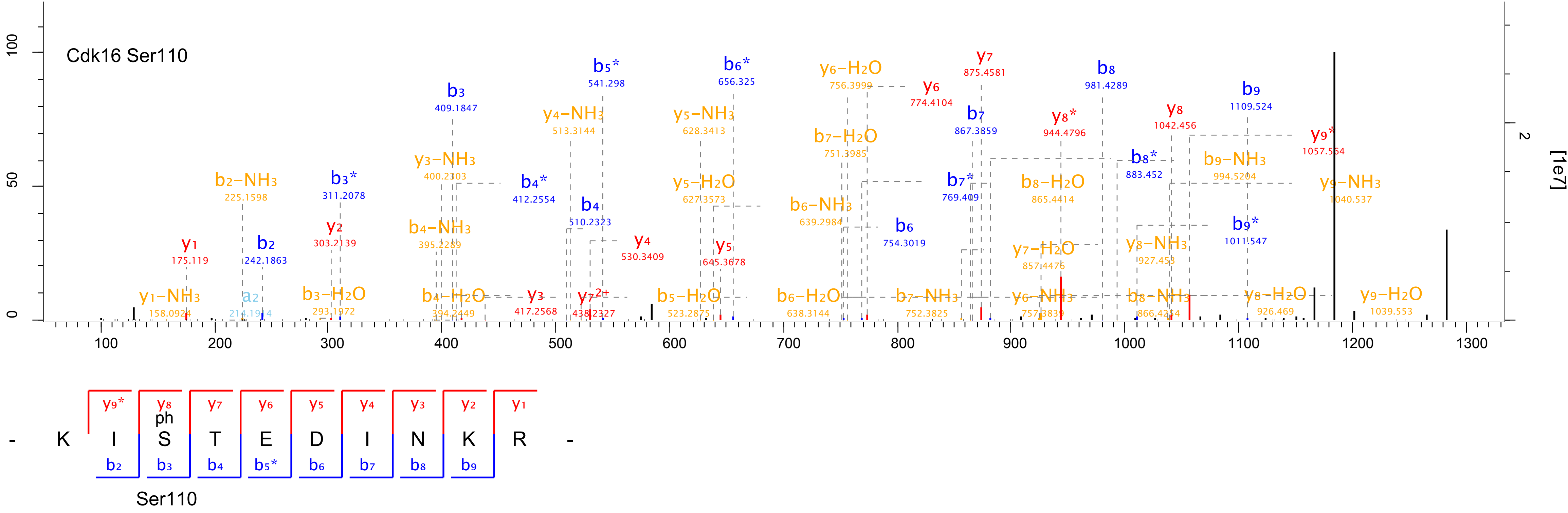

Raw file	Scan	Method	Score	m/z	Gene names
F_20141230_JRWu_ZZZheng_Pho_2_UM2	31463	FTMS; HCD	174.76	1025.54	Cdk16

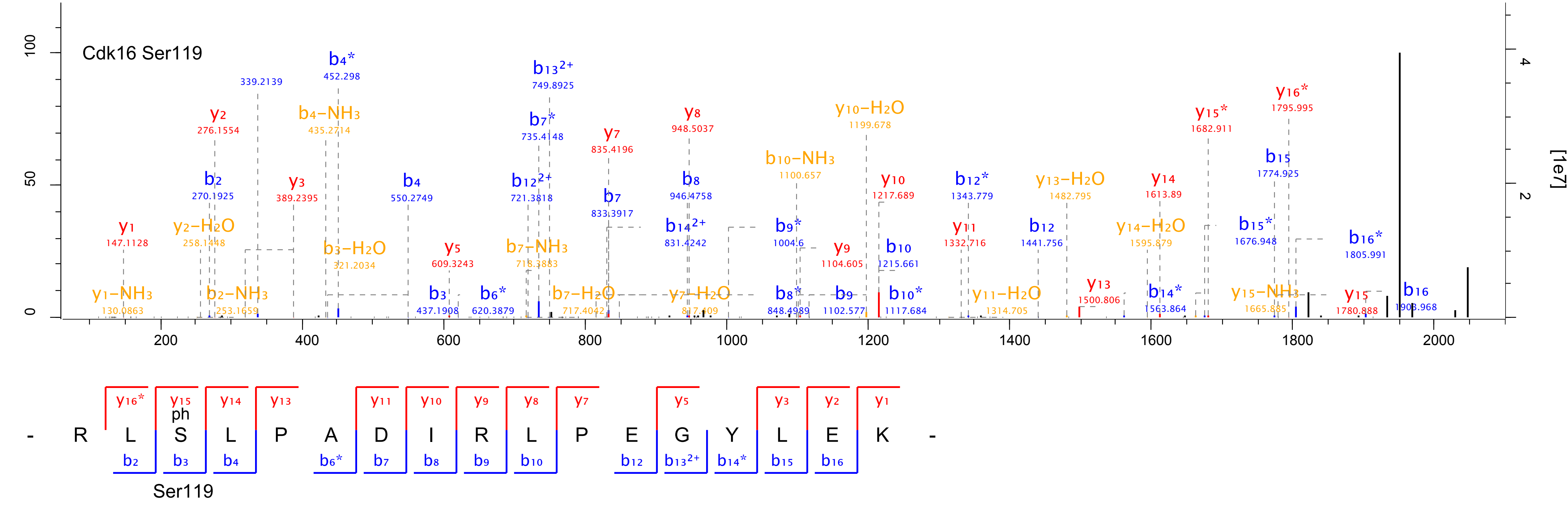

Raw file	Scan	Method	Score	m/z	Gene names
F_20141230_JRWu_ZZZheng_Pho_2_UM2	27931	FTMS; HCD	272.23	841.44	Cdk16

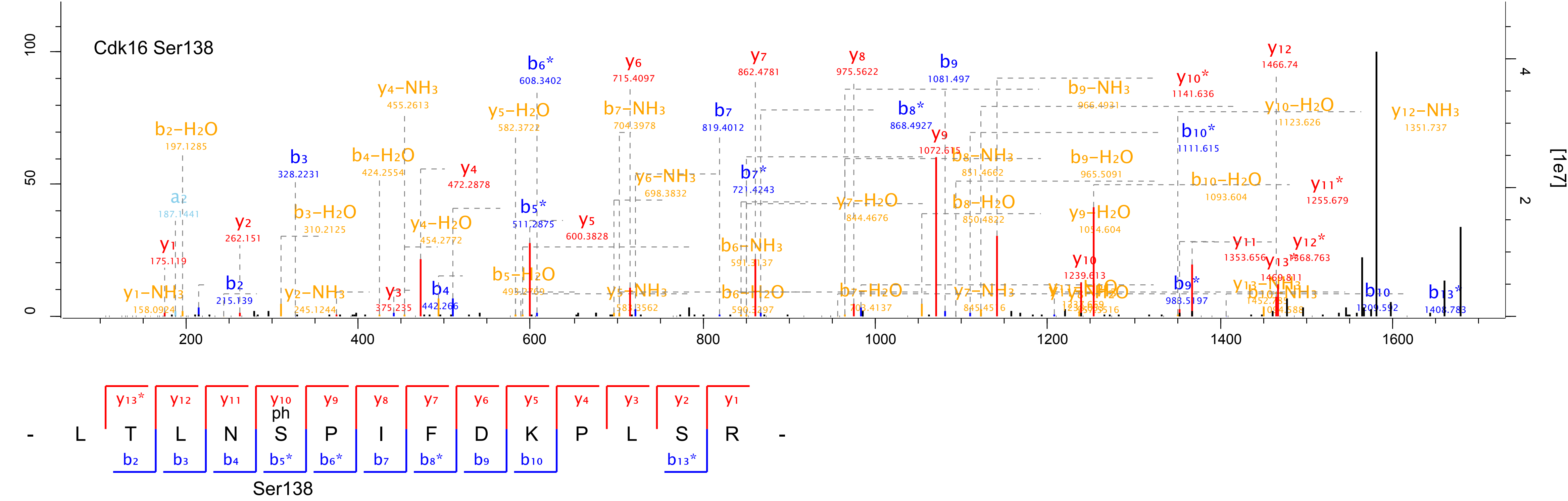

Raw file	Scan	Method	Score	m/z	Gene names
F_20141230_JRWu_ZZZheng_Pho_2_UM2	24766	FTMS; HCD	98.68	639.65	Cdk16

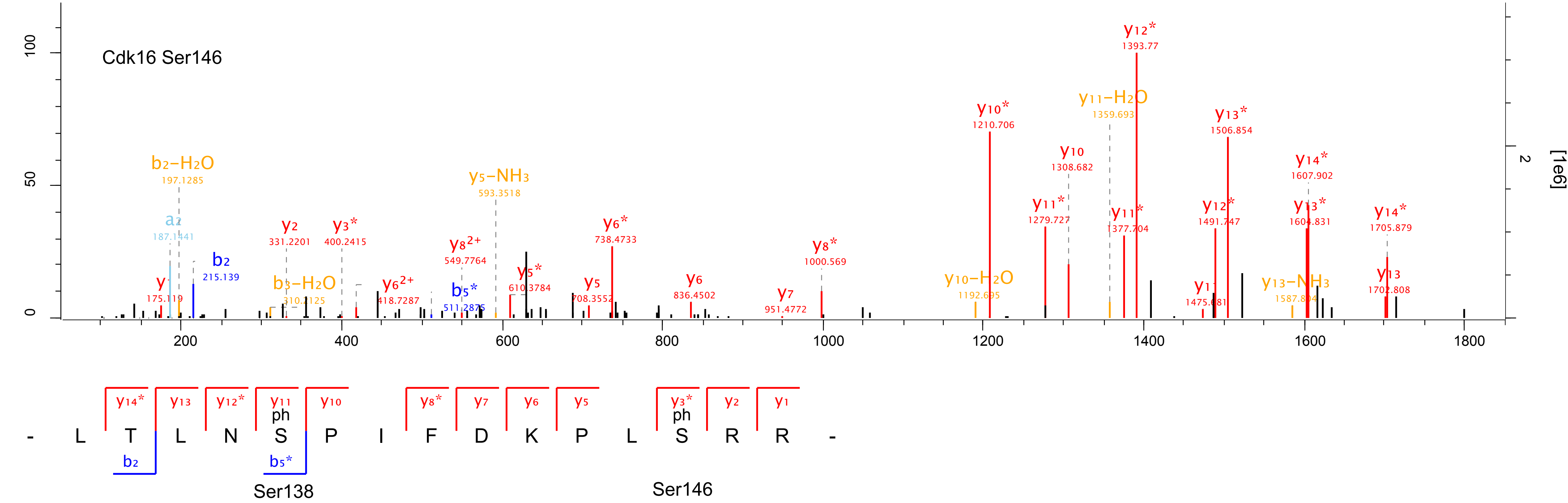

Raw file	Scan	Method	Score	m/z	Gene names
F_20141230_JRWu_ZZZheng_Pho_1_UM1	25107	FTMS; HCD	264.44	636.82	Cdk16

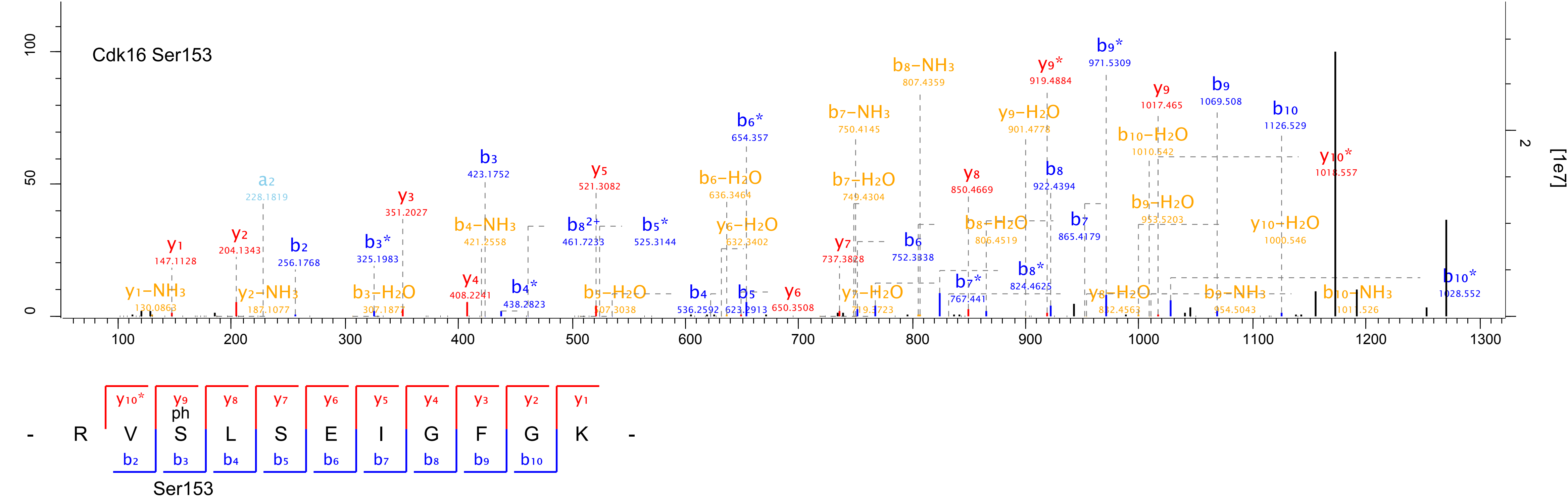

Raw file	Scan	Method	Score	m/z	Gene names
F_20141219_JRWu_ZZZheng_Pho_2_double-2	21127	FTMS; HCD	135.1	558.77	Cdk16

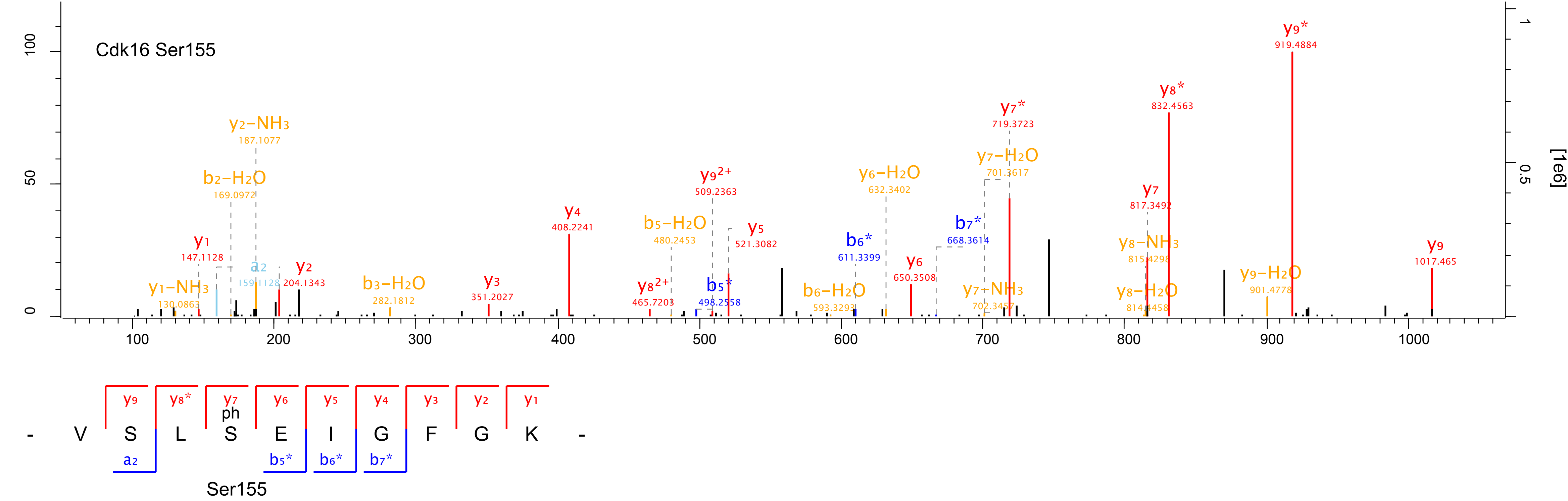

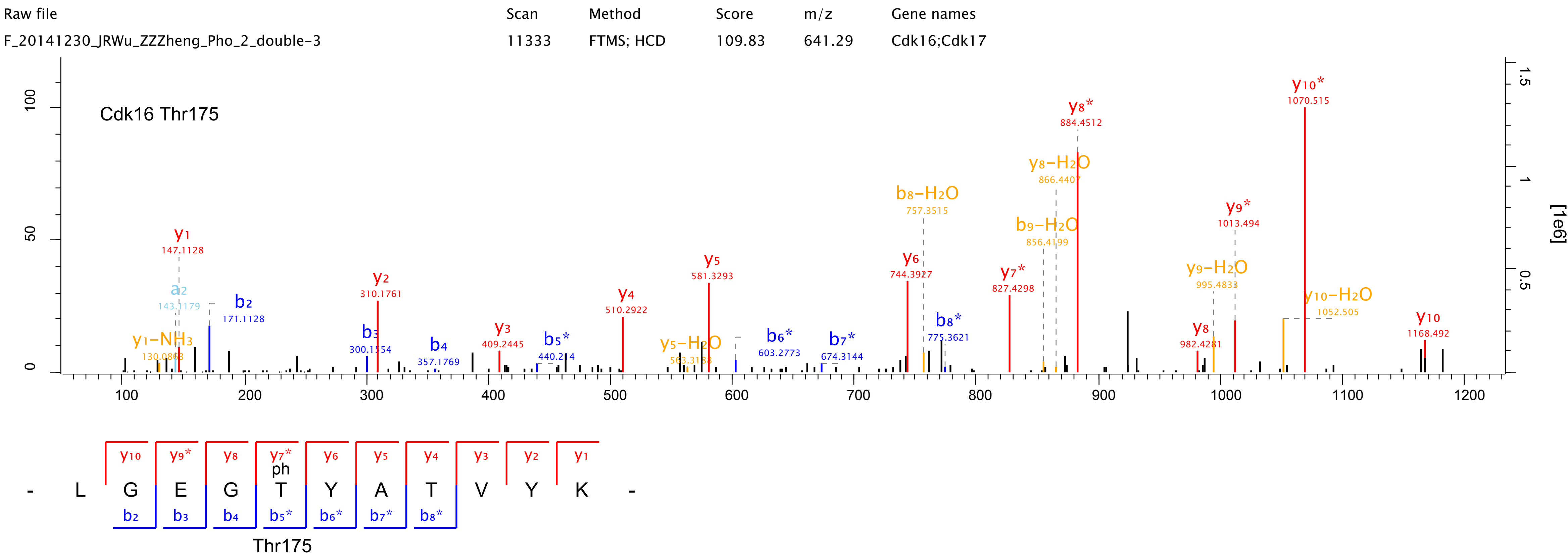

Raw file
F_20141230_JRWu_ZZZheng_Pho_2_double-3
Scan
11333
Method
FTMS; HCD
Score
109.83
m/z
641.29
Gene names
Cdk16;Cdk17
Cdk16 Thr175
y1 147.1128
a2 143.1179
b2 171.1128
y1-NH3 130.0863
b3 300.1554
y2 310.1761
b4 357.1769
y3 409.2445
b5* 440.214
y4 510.2922
y5 581.3293
y5-H2O 563.3188
b6* 603.2773
b7* 674.3144
y6 744.3927
b8-H2O 757.3515
b8* 775.3621
y7* 827.4298
b9-H2O 856.4199
y8-H2O 866.4407
y8* 884.4512
y8 982.4281
y9-H2O 995.4833
y9* 1013.494
y10-H2O 1052.505
y10* 1070.515
y10 1168.492
[1e6]
- L G E G T Y A T V Y K -
ph
Thr175

Raw file	Scan	Method	Score	m/z	Gene names
F_20141230_JRWu_ZZZheng_Pho_2_UM2	33917	FTMS; HCD	96.71	1185.15	Cdk16

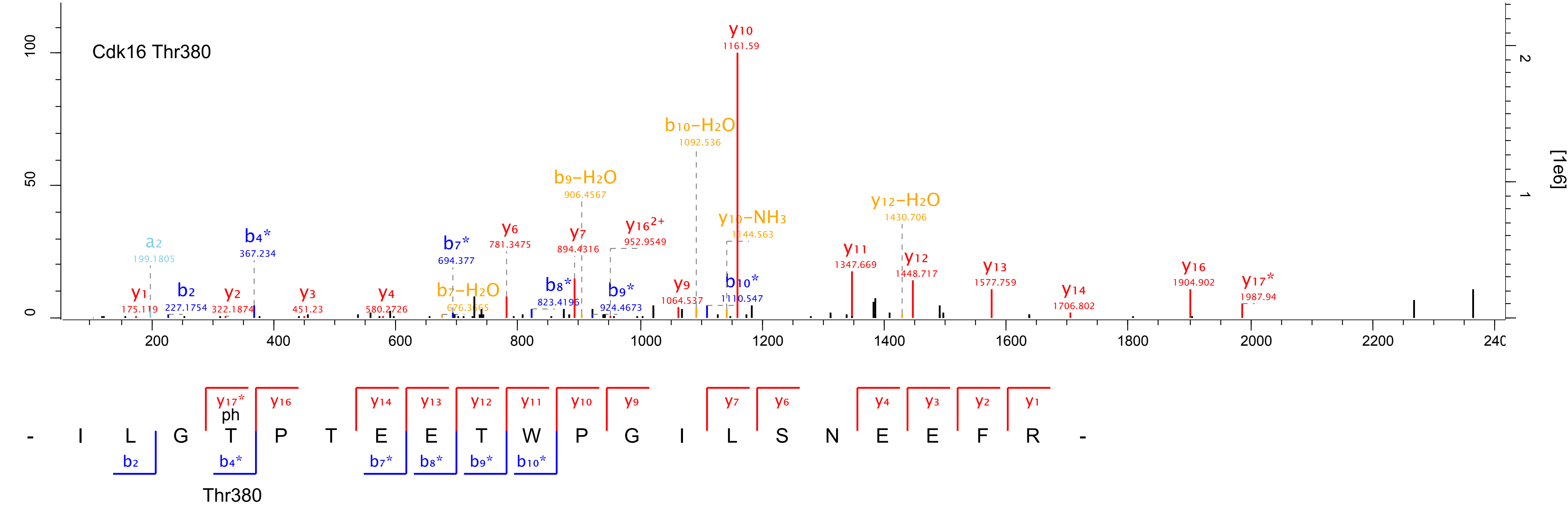

Raw file	Scan	Method	Score	m/z	Gene names
F_20141230_JRWu_ZZZheng_Pho_2_double-2	22960	FTMS; HCD	107.88	1185.56	Cdk16

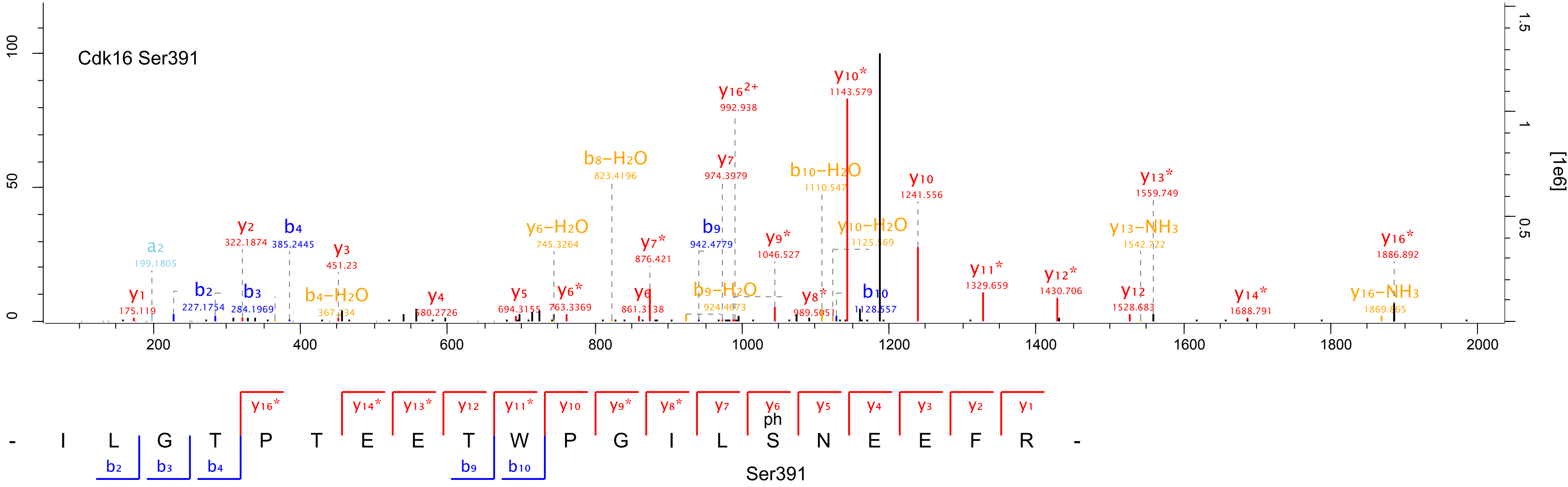

Raw file	Scan	Method	Score	m/z	Gene names
F_20141230_JRWu_ZZZheng_Pho_2_double-2	9204	FTMS; HCD	129.43	691.32	Cdk16

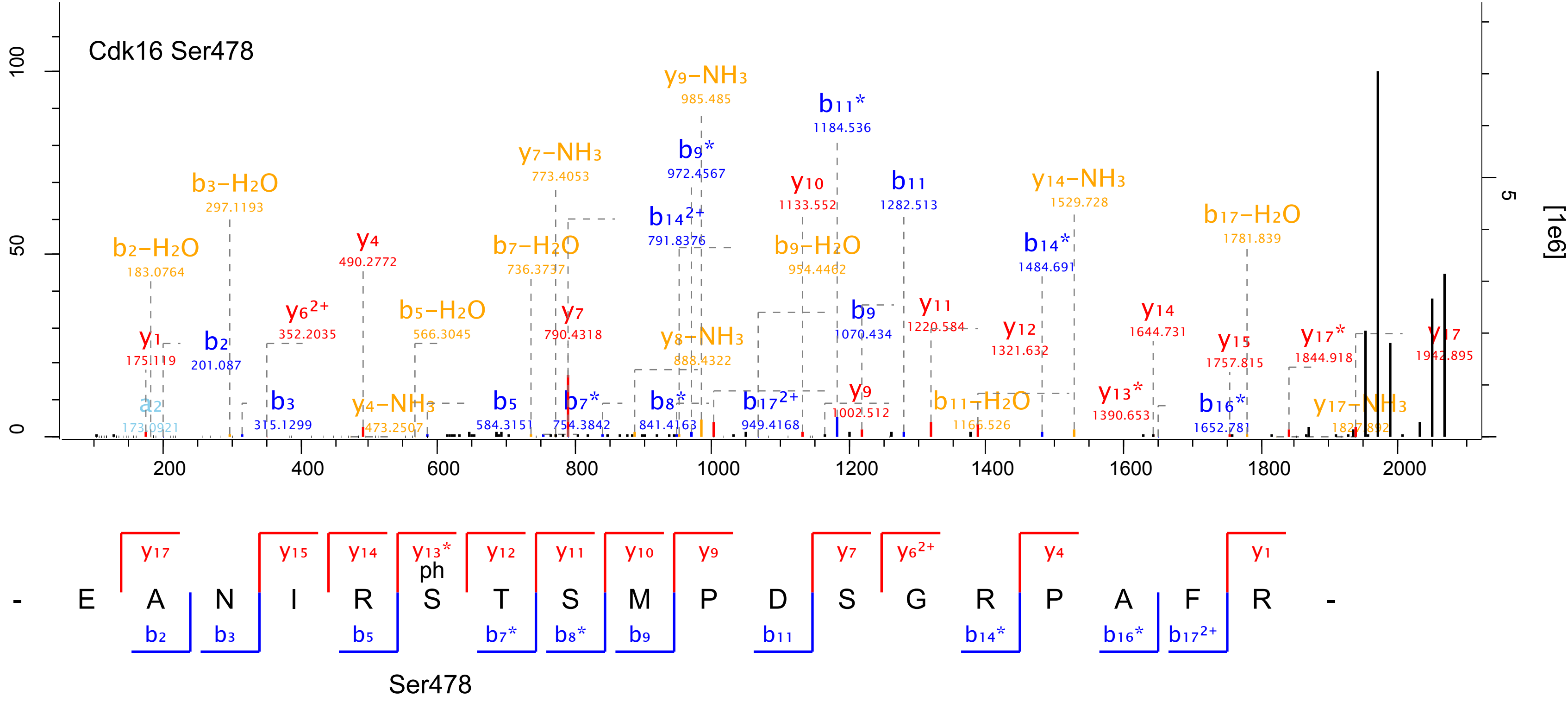

Raw file	Scan	Method	Score	m/z	Gene names
F_20141219_JRWu_ZZZheng_Pho_1_single-1	8891	FTMS; HCD	145.1	497.22	Cdk16

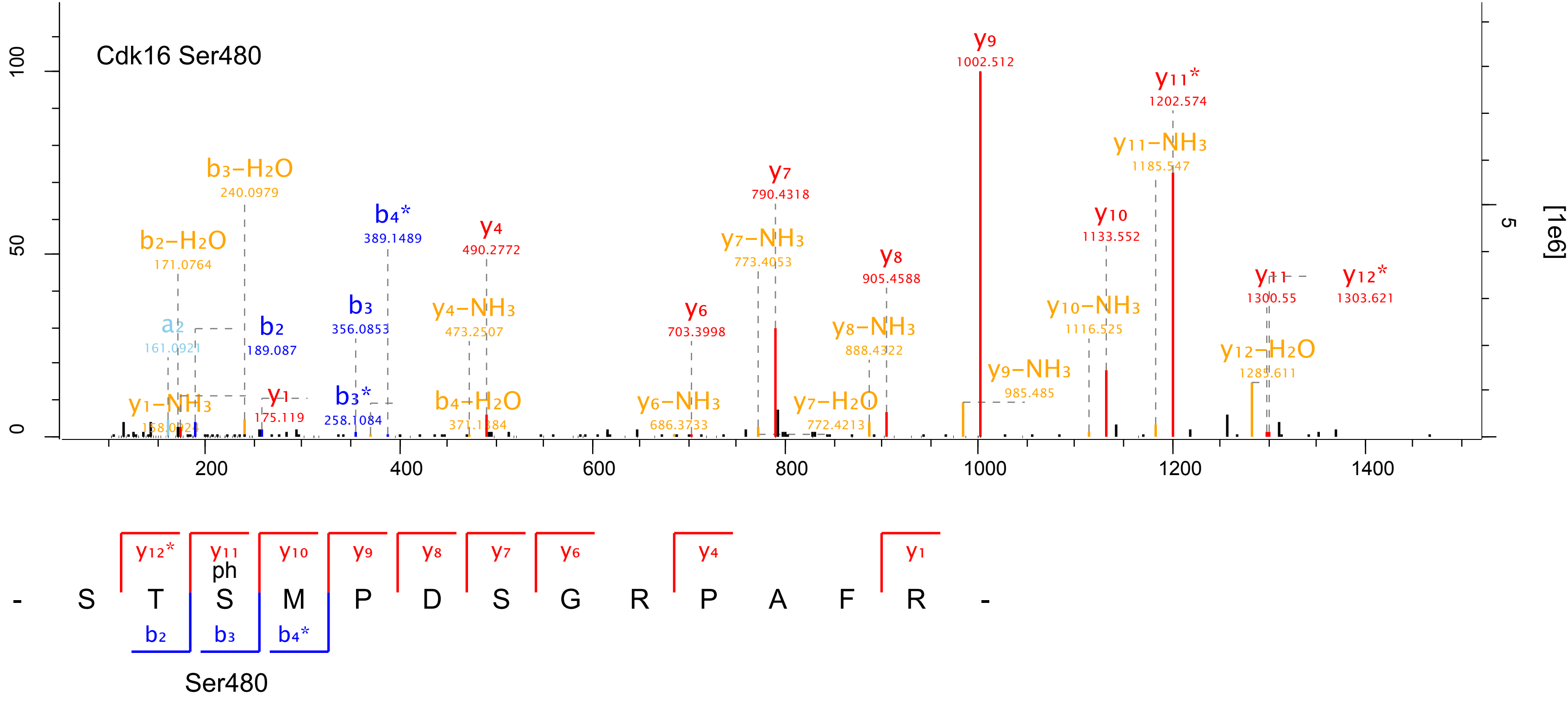

Supplement: S1 Dataset — (PDF) [file pgen.1005485.s011.pdf]
