# Supplementary figures and images for: Correction: CCNYL1, but Not CCNY, Cooperates with CDK16 to Regulate Spermatogenesis in Mouse
Source: PLoS Genet. 2019 Mar 4;15(3):e1008021. doi: 10.1371/journal.pgen.1008021 (PMC6398826; doi:10.1371/journal.pgen.1008021)

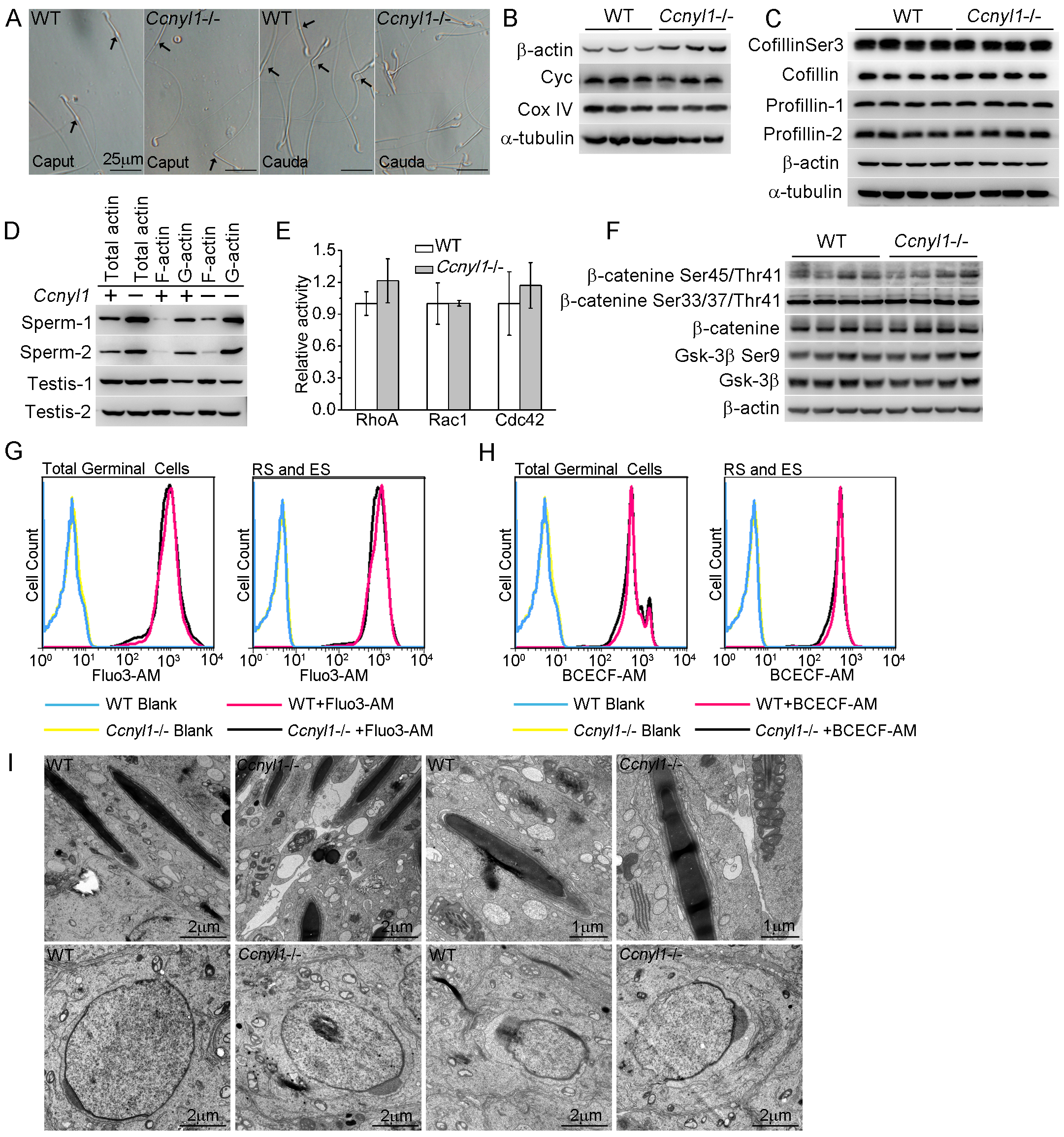

Supplement: S5 Fig — (A) DIC images of spermatozoa collected from caput and cauda epididymidis of adult WT and Ccnyl1-/- mice. Black arrow: cytoplasmic droplets, Scale bar: 25 μm. (B) Measurement of β-actin, Cyc (Cytochrome C) and Cox IV (Cytochrome c Oxidase Subunit IV) protein levels of WT and Ccnyl1-/- spermatozoa (n = 3 per group), with α-tubulin serving as loading control. (C) Measurement of Cofilin, p-ser3-Cofilin1, Profilin1, Profilin-2 and β-actin protein levels in testis of WT and Ccnyl1-/- mice (n = 4 per group), with α-tubulin serving as loading control. (D) Isolation of F-actin and G-actin of WT and Ccnyl1-/- spermatozoa/testes (n = 2 per group). The F-actin fraction and G-actin fraction were dissolved in an equal volume of buffers, and their contents were examined by western blot. (E) RhoA, Rac1 and Cdc42 activities were measured in testicular lysates of WT and Ccnyl1-/- mice (n = 4 per group). The activity was normalized to that of WT mice, which was defined as 1.0. Data are presented as mean ± SEM. (F) Western blotting analysis of p-Ser45/Thr41 β-catenine, p-Ser33/Ser37/Thr41 β-catenine, β-catenine, p-Ser9-Gsk3β and Gsk3β protein levels in testes of WT and Ccnyl1-/- mice (n = 4 per group), with β-actin serving as loading control. (G-H) Measurements of intracellular Ca2+ and pH levels of germ cells. Mouse germ cells were isolated and co-stained with Hoechst 33342, PI, and (G) Fluo3-AM (Ca2+ probe, 1 μM) or (H) BCECF-AM (pH probe, 0.05 μM). PI staining was used to exclude the dead cells, while Hoechst 33342 was used to assign the germ cells into different populations according to their DNA content. 300,000 total cells from each group were examined by FACS analysis. RS: round spermatids; ES: elongating and elongated spermatids. (I) TEM images of seminiferous tubules obtained from testes of adult WT and Ccnyl1-/- mice. (TIF) [file pgen.1008021.s001.tif]
